# Dietary cholesterol impairs cognition via gut microbiota-derived deoxycholic acid in obese mice

**DOI:** 10.1080/19490976.2025.2537753

**Published:** 2025-07-28

**Authors:** Yan Liu, Zhangtie Wang, Yansong Zhang, Tian Zhao, Qingjun Zhang, Weisu Huang, Baiyi Lu

**Affiliations:** a College of Biosystems Engineering and Food Science, Key Laboratory for Agro-Products Nutritional Evaluation of Ministry of Agriculture and Rural Affairs, Zhejiang University, Hangzhou, China; b Department of Applied Technology, Zhejiang Econ & Trade Polytech, Hangzhou, China

**Keywords:** Dietary cholesterol, gut microbiota, bile acid, deoxycholic acid, cognitive impairment

## Abstract

Dietary cholesterol is often found in a high-fat diet (HFD) and excessive intake is harmful to cognitive function. The gut microbiome constitutes an environmental factor influenced by diet, which regulates cognitive function via the gut-brain axis. The present study explored the role of dietary cholesterol in HFD-induced cognitive impairment and the participation of the gut microbiota and metabolites. Here, we found that dietary cholesterol promoted cognitive impairment in HFD-fed mice, which was associated with an increase in gut microbiota containing 7α-dehydroxylase, including *Lachnospiraceae* bacterium, *Dorea* sp. *Clostridium* sp. and elevated levels of deoxycholic acid (DCA) in the hippocampus. Upon dietary cholesterol intake, the activity of gut microbiota in mice to produce DCA is increased. Fecal microbiota transplantation confirmed that the cognitive impairment-promoting process was driven by gut microbiota. Reducing circulating bile acid levels with cholestyramine improved cognitive decline in mice, whereas hippocampal administration of DCA worsened cognitive function. Pharmacological inhibition of hippocampal apical sodium bile acid transporter reduces neuronal DCA accumulation and improves neuronal apoptosis as well as cognitive impairments in mice. Overall, this study revealed that dietary cholesterol promotes HFD-induced cognitive impairment by inducing the production of DCA through gut microbiota metabolism.

## Introduction

Dietary cholesterol, found in animal-based foods such as meats, eggs, and dairy products, is a vital nutritional component that provides essential lipids for the human body.^1,2^ While recommendations limiting dietary cholesterol intake have been removed from the dietary guidelines of several countries, the hazards of excessive cholesterol consumption remain noteworthy.^3^ In recent years, associations between dietary cholesterol intake and risks of cardiovascular and hepatic diseases have been consistently reported.^4,5^ The lipotoxicity induced by excessive cholesterol intake may also contribute to the pathogenesis of multiple diseases, including diabetes, chronic kidney disease, and Alzheimer’s disease (AD), among others.^6^

Over the past two decades, ongoing research has focused on the relationship between cholesterol and cognitive decline. Previous cross-sectional research has demonstrated a strong link between increased dietary cholesterol consumption and reduced cognitive performance.^7,8^ A recent study indicated that APOE4+ carriers who consumed more cholesterol exhibited poorer lipid profiles, correlating with a higher risk of developing dementia and cognitive impairment.^9^ The cognitive impairment effects of dietary cholesterol have also been observed in mice.^10,11^ A study in transgenic mice revealed that cholesterol accumulation in neuronal mitochondria drives neurotoxicity, implying that endogenous neuronal cholesterol loading may impair cognition through neurotoxic mechanisms.^12^ However, exogenous cholesterol (i.e., dietary-derived cholesterol) appears to have difficulty directly affecting neurological functions, as cholesterol-carrying lipoproteins cannot cross the intact blood-brain barrier (BBB).^6^

Bile acids (BAs), as gut microbial metabolites modulated by dietary cholesterol, can reach brain and influence neurological functions. Dietary cholesterol can be absorbed into the circulation through the small intestine after being incorporated into bile salt micelles. However, with an increase in dietary cholesterol intake, the absorption rate of cholesterol in the human body gradually decreases,^13^ resulting in more cholesterol reaching the large intestine. Cholesterol influences the gut microbiome, thereby affecting the composition of gut microbial metabolites, includingbile acids (BAs).^1^ For example, Gao et al. found that dietary cholesterol promotes nonalcoholic steatohepatitis in obese mice by increasing serum cholic acid (CA) and deoxycholic acid (DCA) levels through the growth of *Bacteroides*, *Clostridium*, and *Lactobacillus*, which contain bile salt hydrolase (BSH).^14^ On the other hand, Zhang et al. observed that dietary cholesterol regulates the gut microbiota, leading to an increase in taurocholic acid and a decrease in 3-indolepropionic acid, thereby promoting fatty liver-associated liver cancer.^15^ These metabolites gain access to the brain from the periphery circulation either by passive diffusion across the BBB or through specific transporters like the apical sodium-bile acid transporter (ASBT), potentially modulating brain physiological functions. Some cholesterol-related metabolites, especially BAs, have been shown to correlate with cognitive function. A study based on 1464 subjects reported that, compared with controls, AD patients typically exhibit reduced levels of primary BAs in their serum, as well as elevated levels of secondary BAs.^16^ It was also observed that in postmortem brain from AD patients, the secondary BAs were greater than those in the controls.^17^

Long-term consumption of a high-fat diet (HFD) impairs cognitive function.^18^ Apart from eggs, high-cholesterol foods are typically animal-based high-fat products such as organ meats, fatty meats, and dairy products. Critical questions persist regarding: (a) how dietary cholesterol modulates HFD-induced cognitive impairment, and (b) whether gut microbial metabolites serve as mediators in this pathway. Thus, this study aimed to investigate the role and molecular mechanisms underlying the impact of dietary cholesterol on HFD-induced cognitive impairment.

## Materials and methods

### Animal experiments

Eight-week-old male C57BL/6J mice were purchased from Charles River Laboratories (Jiaxing, Zhejiang, China). All mice were housed in a controlled environment adhering to specific pathogen-free standards, with a 12-hour light/dark cycle, a temperature range of 20–24°C, and humidity levels maintained between 40–60%. Mice had free access to food and water. Before the commencement of any experimental procedures, all mice were allowed a minimum acclimatization period of one week.

Animal experiment 1: To examine the effects of dietary cholesterol on cognitive function in HFD-fed mice, animals were supplemented with 0.2%, 0.5%, or 1% cholesterol-enriched HFD, followed by comprehensive behavioral assessment of cognitive performance. The mice were randomly assigned to five groups (*n* = 10): the Control group (chow diet, Trophic Feed, China, #TP23102), HFD group (45% calories from fat, Trophic Feed, China, #TP23100), HFD + 0.2%Chol group (HFD plus 0.2% cholesterol), HFD + 0.5%Chol group (HFD plus 0.5% cholesterol), and HFD + 1%Chol group (HFD plus 1% cholesterol). A 0.2% cholesterol dose has been extensively applied in research investigating the impact of dietary high-cholesterol on mice.^15,19^ Using the body surface area conversion formula (mouse dose × 0.081 = human dose), and assuming a mouse weighs 25 g and consumes 3 g of feed, this cholesterol dose is roughly equivalent to a daily intake of 1166 mg for a 60 kg human.^20^ Accounting for differences in cholesterol absorption rates between mice and humans, this dose corresponds to about 580 mg of cholesterol per day in humans, which is within the range of a typical adult diet.^21,22^ As the mice gain weight during the study, the equivalent dose in humans decreases, gradually approaching the average daily cholesterol intake in a standard human diet. To investigate the dose-response relationship between dietary high-cholesterol and cognitive function, additional doses of 0.5% and 1% were included. The ingredients in the Control diet and HFD were showed in Supplementary information 1 Table S1. Each group of mice received the corresponding diet for six months. Feces were collected during the last month. Then, cognitive tests were carried out. After that, the anesthetized mice were humanely euthanized through cervical dislocation. Serum, tissues, and colonic contents were harvested and immediately frozen at −80°C for further analysis. Some of murine brain tissue was immersed in paraformaldehyde for subsequent Hematoxylin-eosin (H&E) staining. This experiment was conducted in two batches. The first batch of hippocampal samples was utilized for transcriptome analysis and H&E staining. The second batch of hippocampal samples was subjected to Western blot, bile acid composition, cholesterol level, and RT-qPCR analysis.

Animal experiment 2: To investigate the isolated effects of dietary cholesterol on cognitive function in mice, animals were maintained for six months on control diets supplemented with 0.2%, 0.5%, or 1% (w/w) cholesterol. Cognitive performance was then comparatively assessed across dosage groups. The mice were randomly assigned to four groups (*n* = 10): the Control group (chow diet), 0.2%Chol group (chow diet plus 0.2% cholesterol), 0.5%Chol gourp (chow diet plus 0.5% cholesterol), and 1%Chol group (chow diet plus 1% cholesterol). Each group of mice received the corresponding diet for six months. Then, cognitive tests were carried out. After that, the anesthetized mice were humanely euthanized through cervical dislocation. Tissues and colonic contents were harvested and immediately frozen at −80°C for further analysis.

Animal experiment 3: To investigate the role of gut microbiota in mediating dietary cholesterol-induced cognitive impairment, recipient mice maintained on HFD underwent fecal microbiota transplantation (FMT) from three donor groups: Control diet-fed mice, HFD-fed mice, and HFD + 0.2% cholesterol-fed mice. Cognitive function was comparatively assessed post-FMT. The mice that received FMT were randomly assigned to three groups (*n* = 10): the Control→HFD group (mice received fecal microbiota from the Control group), HFD→HFD group (mice received fecal microbiota from the HFD group), and HFD + 0.2%Chol→HFD group (mice received fecal microbiota from the HFD + 0.2%Chol group). Fresh feces were collected from the Control, HFD, and HFD + 0.2%Chol groups and stored in PBS supplemented with 20% glycerol at − 80°C. Before the FMT, the recipient mice were administered antibiotics in their drinking water containing ampicillin (1 mg/mL), vancomycin (5 mg/mL), neomycin (10 mg/mL), and metronidazole (10 mg/mL) for 10 days to deplete their gut microbiota.^23^ Saccharin sodium was added to the antibiotic solution to mask the taste of the antibiotics and prevent dehydration in mice. Moreover, fecal samples were collected from the mice before and after antibiotic treatment to assess the efficacy of antibiotic-induced gut microbiota depletion and subsequent FMT in establishing gut microbiota colonization. The fecal microbiota suspension was prepared according to the following method.^24^ First, frozen fecal samples were thawed in a water bath at 37.5°C for 5 min. The feces were then homogenized in pre-reduced PBS (feces/PBS = 1:10, w/w) and were centrifuged at 600 × g for 3 min to remove insoluble materials. The obtained fecal microbiota suspension was centrifuged at 15,000 × g for 5 min to obtained bacterial pellets. Then, the bacterial pellets were re-suspended and transplanted into recipient mice. The oral gavage dose of fecal suspension for the mice was 10 mL/kg body weight. Each mice received FMT once every five days for six months. Then, cognitive tests were carried out. After that, the anesthetized mice were humanely euthanized through cervical dislocation. Serum and tissues were collected for further analysis.

Animal experiment 4: To confirm the causal relationship between gut microbiota and dietary cholesterol-induced cognitive impairment, recipient mice maintained on HFD + 0.2% cholesterol received FMT from three donor cohorts: Control diet-fed mice, HFD-fed mice, and HFD + 0.2% cholesterol-fed mice. The mice that received FMT were randomly assigned to three groups (*n* = 10): the Control→HFD +0.2%Chol group (mice received fecal microbiota from the Control group), HFD→HFD +0.2%Chol group (mice received fecal microbiota from the HFD group), and HFD + 0.2%Chol→HFD +0.2%Chol group (mice received fecal microbiota from the HFD + 0.2%Chol group). Mice received FMT for 4 months. The fecal microbiota preparation and antibiotic pretreatment procedures followed the identical protocol established in Animal Experiment 3.

Animal experiment 5: To investigate the contribution of dietary cholesterol-induced BAs accumulation to cognitive impairment, 2% cholestyramine (CHY) was administered to HFD + 0.2% cholesterol-fed mice. This intervention aimed to reduce cerebral bile acid levels, enabling comparative assessment of cognitive function following bile acid depletion. The mice were assigned to the HFD + 0.2%Chol and HFD + 0.2%Chol+CHY (HFD plus 0.2% cholesterol and 2% CHY) groups. The 2% CHY in the diet was used as a bile acid sequestrant to reduce the total bile acid in the serum.^25^ Each group of mice received the corresponding diet for six months. Then, cognitive tests were carried out. After that, the anesthetized mice were humanely euthanized through cervical dislocation. Serum and tissues were collected for further analysis.

Animal experiment 6: To investigate the direct effects of DCA on cognitive function in HFD-fed mice, DCA was administered via intracerebroventricular infusion. Cognitive performance was then comparatively assessed between DCA-supplemented and vehicle-treated HFD-fed cohorts. The mice were assigned to the HFD and HFD+DCA groups. In Animal experiment 1, the hippocampal DCA concentration in mice fed an HFD + 1%Chol diet was found to be on average 0.498 pM/mg tissue higher than that in the HFD group. Considering a hippocampal average weight of 40 mg in mice, it was calculated that dietary cholesterol led to a 0.0199 nM increase in DCA levels within the hippocampus of HFD-fed mice. Therefore, the daily dose of DCA (#D2510, Sigma‒Aldrich, MO, USA) administered to HFD-fed mice is set at 0.0199 nM. Osmotic minipumps (#2006, Alzet, USA) were used to infuse DCA or control solution into the hippocampus of mice at a flow rate of 0.15 μL/h for one month. After five months on an HFD, minipumps were implanted into the mice. DCA was dissolved in DMSO, diluted in artificial cerebrospinal fluid, and then preloaded into the pumps. The pumps were soaked in sterile 0.9% saline at 37°C for 48 h. Mice were anesthetized and mounted on a stereotaxic apparatus. Cannulas were bilaterally implanted into the hippocampus (relative to bregma: A/P = −2 mm; L/M = ±1.5 mm; D/V = −1.5 mm). The pump body was placed in a pocket between the skin and muscle of the scruff through an incision, which was then sutured with dissolvable stitches. A local antibiotic was applied to the surgical site, and the mice were allowed to recover in clean cages on a warm heating pad until fully awake. Mice implanted with minipumps received carprofen (5 mg/kg) pre- and post-operatively to alleviate pain. After the one-month administration period, cognitive tests were carried out. After that, the anesthetized mice were humanely euthanized through cervical dislocation. Tissues were collected for further analysis.

Animal experiment 7: To investigate hippocampal ASBT’s contribution to DCA-mediated neurotoxicity in obesity, the ASBT inhibitor, GSK2330672 (GSK), was administered via intracerebroventricular infusion. Cognitive function was comparatively assessed between GSK-treated and vehicle-control HFD mice following ASBT inhibition. The mice were divided into four groups: HFD, HFD+GSK, HFD+DCA, and HFD+DCA+GSK. After 5 months and 1 week of HFD feeding, minipumps were implanted into the mice as described in Animal Experiment 5. The hippocampus of the mice from the HFD, HFD+GSK, HFD+DCA, and HFD+DCA+GSK groups was infused with control solution, GSK, DCA, and a combination of DCA and GSK, respectively. The daily dose of DCA administered to the mice was 0.0199 nM, as described in Animal Experiment 5. Based on a previous study where 1 mg/kg of GSK was administered for two weeks to inhibit ASBT,^26^ and considering the average hippocampal weight of 40 mg in mice in our study, 0.04 µg of GSK was infused into the hippocampus daily. After a 3-week treatment period, cognitive function was assessed through behavioral tests. Following the tests, the mice were humanely euthanized under anesthesia by cervical dislocation, and tissues were collected for further analysis.

Animal experiment 8: To investigate the role of hippocampal farnesoid X receptor (FXR) in mediating dietary cholesterol-induced cognitive impairment, adeno-associated virus (AAV)-mediated FXR knockdown was performed in the hippocampus. Cognitive function was comparatively assessed between FXR-knockdown and control-vector mice on cholesterol-enriched diets. The mice were assigned to the HFD+AAV-Control, HFD + 0.2%Chol+AAV-Control, HFD+AAV-*Nr1h4*, and HFD + 0.2%Chol+AAV-*Nr1h4* groups. After 4 months of HFD or HFD + 0.2% cholesterol administration, the mice were anesthetized, and the viruses were delivered via syringe at a rate of 50 nL/min. The needles were left undisturbed for an extra 10 min before removal. The viruses were administered into both hemispheres of the hippocampus (relative to the bregma: A/P = −2 mm; L/M=±1.5 mm; D/V = −1.5 mm). A volume of 0.5 μL of the *Nr1h4* overexpression or control virus (1 × 10^12^ v.g./mL) was injected into each side of the hippocampus. The incision was sutured with dissolvable sutures, and the mice were administered carprofen (5 mg/kg) pre- and post-operatively to alleviate pain. The viruses were obtained from WZ Biosciences, Inc. (Shandong, China). Cognitive function tests were conducted 2 months after the injections. After that, the anesthetized mice were humanely euthanized through cervical dislocation. Tissues were collected for further analysis.

Animal experiment 9: To investigate the role of hippocampal caspase-3 in mediating dietary cholesterol-induced cognitive impairment, the caspase-3 inhibitor Z-DEVD-FMK (ZDF) was administered via intracerebroventricular infusion. Cognitive function was comparatively assessed between caspase-3-inhibited and vehicle-treated cohorts. The mice were assigned to the HFD, HFD + 0.2%Chol, HFD+ZDF, and HFD + 0.2%Chol+ZDF groups. In previous studies, each mouse received 1 μL of 174 μM ZDF per side of the hippocampus (a total of 348 nM ZDF for a hippocampus), which effectively inhibited caspase 3 activation.^27^ Therefore, osmotic minipumps were used to continuously infuse 348 nM ZDF (#Z872464, Macklin, Shanghai, China) into the hippocampus daily. ZDF was dissolved in DMSO, further diluted in artificial cerebrospinal fluid, and preloaded into the pumps. The method of pump implantation followed the procedure described in Animal Experiment 5. The pumps were implanted after the mice had been on their corresponding diet for five months, and cognitive function was tested one month after pump implantation. After that, the anesthetized mice were humanely euthanized through cervical dislocation. Tissues were collected for further analysis.

### Cognitive function tests

Y-maze: The Y-maze test was employed to evaluate spontaneous alternation behavior.^28^ The experimental setup featured a maze with three arms, each measuring 35 cm in length, 15 cm in height, and 5 cm in width. These arms converged at equal angles and were arranged in a manner that allowed for successive entries into overlapping triplet configurations (Wuhan Ruyi Organic Glass Products Factory, Hubei, China). Initially, mice were placed at the center of the apparatus and given 8 min to explore. During this time, the total number of arm entries and spontaneous alternations were recorded. The percentage of alternations was calculated as the ratio of the actual number of alternations to the total possible alternations (defined as the total number of arm entries minus 2) × 100%. Differences in total arm entries between groups were compared to determine whether variations in spontaneous alternation rate were influenced by total arm entries.

Novel object recognition (NOR): The NOR experiment was conducted according to a previously reported method.^29^ The NOR experiment lasted for 3 d, encompassing a day for habituation, another for training, and a final day dedicated to testing. During the habituation phase, mice were placed in a 40 × 40 × 40 cm open field, where they had unrestricted access to explore the space for 5 min. Following this, each mouse was moved to a holding cage, and this procedure was repeated until every mouse from the same home cage had completed the exploration. Finally, all mice were returned to their home cage collectively. After each mouse completed the exploration, the field was cleaned with 70% alcohol. On the training day, two identical plastic objects were symmetrically placed in the center of the field, maintaining equidistance between the objects and the field walls. After 5 min of free exploration by the mice, they were transferred as described above. On the testing day, one of the two identical objects from the training session was substituted with a new object of the same material but differing in color and shape. Mice were given unrestricted access to explore for 5 min. The duration of exploration time dedicated by each mouse to both new and old objects was meticulously documented. The discrimination ratio was calculated as the time spent exploring the new object divided by the total exploration time multiplied by 100%. Differences in total exploration time between groups were compared to determine whether variations in discrimination rate were influenced by total exploration time.

### Biochemical assay and histological examination

Serum low-density lipoprotein cholesterol (LDL-C) and high-density lipoprotein cholesterol (HDL-C) was determined using an LDL-C and HDL-C assay kit (#A113–1–1/A112–1–1, Nanjing Jiancheng, Jiangsu, China), respectively. Total cholesterol levels in serum and hippocampal samples were determined using a Total Cholesterol Assay Kit (#A111–1–1, Nanjing Jiancheng, Jiangsu, China), and protein quantification was performed via the bicinchoninic acid (BCA) method. The cholesterol extraction steps were as follows: Briefly, tissue samples were accurately weighed and homogenized mechanically in ice-cold ethanol at a 1:20 (w/v) ratio. The homogenates were then centrifuged at 2500 × g for 10 min at 4°C, with subsequent collection of supernatants for downstream analysis.

Hepatic protein expressions of CYP7a1, CYP8b1, CYP27a1, and CYP7b1 was measured using the ELISA kits. The following ELISA kits were used: CYP7a1 (#1188) purchased from Meimian Industrial, Jiangsu, China. CYP8b1, CYP27a1, and CYP7b1 were purchased from Nanjing Jiancheng Bioengineering Institute, with catalog numbers H518, H519, and H349, respectively. Total bile acids were determined using a Total Bile Acid Kit (#E003–2–1, Nanjing Jiancheng, Jiangsu, China). Following fixation of the mouse brains in 4% paraformaldehyde, they were subsequently embedded in paraffin and sliced for H&E staining.

### Transcriptome analysis

Total RNA was extracted from hippocampal samples using TRIzol reagent, and its quantity and purity were assessed using a Bioanalyzer 2100. RNA samples demonstrating an integrity number exceeding 7.0 were chosen for the construction of libraries. mRNA was then purified from the total RNA, fragmented, reverse-transcribed into cDNA, ligated with adapters, amplified by PCR, and subjected to paired-end sequencing on an Illumina NovaSeq 6000 platform. The bioinformatics analysis steps are detailed in Supplementary information 2.

### Metagenomic analysis

After DNA was extracted from the fecal samples, it was concentrated and purified. The DNA extract was subjected to fragmentation, achieving an average size of roughly 400 bp through the use of the Covaris M220, followed by construction of a paired-end library using NEXTFLEX Rapid DNA-Seq. Adapters were ligated to the blunt ends of fragments, and paired-end sequencing was conducted on an Illumina NovaSeq 6000. The network analysis was conducted using the iNAP tool.^30^ The bioinformatics analysis steps are detailed in Supplementary information 2.

### Metabolome analysis

After extracting serum metabolites using a combination of methanol and acetonitrile under low-temperature conditions, the resulting supernatant was centrifuged, subjected to nitrogen flow, and subsequently reconstituted for LC-MS/MS analysis. Serum metabolite profiles were analyzed using UHPLC-Q Exactive HF-X coupled with a mass spectrometer operating in both positive and negative ion modes. The raw data were subjected to meticulous preprocessing, metabolite identification was carried out through database searches, and the databases included HMDB, Metlin, and the Majorbio Database. Next, the data were analyzed using the Majorbio cloud platform (https://cloud.majorbio.com). The sample processing steps and UHPLC-MS/MS methodology are detailed in Supplementary information 2.

### Reverse transcription and real-time PCR

Total RNA was obtained from the mouse hippocampus and cells using TRIzol (#G3013, Servicebio, Wuhan, China) according to the manufacturer’s protocols. Reverse transcription was conducted using a SPARKscript II RT Plus kit (#AG0304-B, SparkJade Biotechnology, Shandong, China), and real-time PCR was conducted on a PCR system (QuantStudio3, Thermo Fisher Scientific, Waltham, MA, USA) using SPARKscript II All-in-one RT SuperMix for qPCR (#AG0305-B, SparkJade Biotechnology, Shandong, China) according to the manufacturer’s protocols. All primers were synthesized by Tsingke Biotechnology (Hangzhou, Zhejiang, China). The primers employed for gene amplification are detailed in Table S2 of the supplementary information 1. Gene expression levels were determined via the 2^−ΔΔCt^ method and subsequently normalized to *GAPDH* expression.

### Western blot

Hippocampal samples were lysed using RIPA buffer to which a mixture of protease inhibitors was added.^31^ After slowly grinding the sample on ice, the tissue lysate was centrifuged at 12,000 × g for 5 min at 4°C. The protein levels in the supernatant were normalized utilizing the BCA assay. The supernatant collected was combined with the SDS-PAGE sample loading buffer and heated at 98°C in a dry bath for 5 min, followed by storage at − 80°C for further analysis. The protein samples were separated using 10 or 12% SDS‒PAGE and blotted onto a PVDF membrane. Following the blocking step using 5% skim milk, the membrane was incubated with the primary antibody at 4°C for an overnight period, and this was succeeded by treatment with the secondary antibody (goat anti-rabbit IgG HRP, Affinity, #AF7021, 1:3000). The primary antibodies used were anti-Tubulin (Proteintech, #11224–1-AP, 1:2000), anti-GAPDH (Affinity, #AF7021, 1:2000), anti-cleaved Caspase 3 (Proteintech, #25128–1-AP, 1:1000), anti-Bax (Proteintech, #50599–2-lg, 1:2000), anti-cleaved Caspase 7 (Proteintech, #27155–1-AP, 1:2000), anti-cleaved Caspase 8 (Proteintech, #13423–1-AP, 1:1000), anti-NeuN (Proteintech, #26975–1-AP, 1:2000), anti-GFAP (Proteintech, #16825–1-AP, 1:2000), anti-IBA-1 (Proteintech, #26177–1-AP, 1:1000), anti-FXR (Bioss, #12867 R, 1:1000), and anti-ASBT (Bioss, #23146 R, 1:1000). The band was exposed to a Super ECL Western Blotting Substrate (#SL1350, Coolaber, Beijing, China) and semi-quantified using a ChemiScope Series 3200 Mini Chemiluminescence Imaging Analysis System (Clinx Science Instruments, Shanghai, China).

### Bile acid composition analysis

The extraction and analysis methods were conducted according to previously reported methods.^32^ Each hippocampal sample and 500 µL of cold methanol were combined in a tube on ice. Then, 100 µL of internal standard at a concentration of 0.01 mg/mL was added. The mixture was homogenized on ice and subsequently centrifuged at 3000 × g for 5 min at 4°C. The supernatant was carefully collected into a new tube. For a second extraction, 400 µL of cold methanol was used. The supernatants from both extractions were then pooled and dried under nitrogen gas. The dried residue was dissolved in a 200 µL solution of methanol and water (1:1, v:v) and subjected to centrifugation at 10,000 × g for 1 min at 4°C. Finally, the obtained supernatant was utilized for analysis. Bile acid compositions were analyzed via UltiMate 3000 Ultra-High-Performance Liquid Chromatography (UHPLC) coupled with a Q Exactive Orbitrap Mass Spectrometer (Thermo Fisher Scientific, Waltham, MA, USA). The analytical conditions were as follows: UHPLC BEH C_18_ column (2.1 × 100 m, 1.7 µm); column temperature: 65°C; column injection volume: 5 µL; solvent system: 0.1% formic acid in water (A), 0.1% formic acid in acetonitrile (B); flow rate: 0.5 mL/min; and elution gradient: initially, for the first 0.5 min, the eluent composition was maintained at 95% A. Subsequently, it was gradually decreased to 75% over 5 min, further reduced to 60% within 10.5 min, and then decreased to 5% over 1.5 min, where it was held for an additional 1.5 min. Finally, the eluent composition returned to 95% A and held constant for 2 min. ESI: negative-ion mode; scan range: 300–600 m/z; stepped nce: 20, 40, and 60. Bile acid compositions were analyzed utilizing scheduled multiple reaction monitoring mode. Data analysis was performed using Thermo Xcalibur Quan Browser software (version 4.1.31.9).

### Bile salt hydrolase (BSH) activity measurement

The BSH activity was measured as previously reported.^33^ Approximately 50 mg of fresh fecal sample was homogenized in 250 μL of PBS, followed by sonication in an ice bath for 90 s at 30 s intervals. The lysate was centrifuged at 15,000 rpm for 30 min at 4°C. The protein concentration was quantified using the BCA assay, and the protein was diluted to 2 mg/mL. A volume of 10 µL of the sample solution was mixed with 180 µL of sodium acetate buffer (pH 5.2) and 10 µL of a 2 mM d4-taurochenodeoxycholic acid (TCDCA) solution, then incubated at 37°C for 30 min. The reaction was halted by immersing the samples in dry ice. Then, 100 µL of methanol was introduced to the mixture, which was then vortexed for 5 min and centrifuged at 15,000 rpm for 20 min at 4°C. The d4-chenodeoxycholic acid (CDCA) content was analyzed using the aforementioned method. BSH activity is defined as the production of d4-CDCA/mg protein/min.

### In vitro *assessment of the ability of fecal microbiota to produce DCA*

The bacterial capacity to transform CA to DCA was measured as previously reported.^34^ A total of 200 mg of mouse feces was suspended in 1 mL of PBS under anaerobic conditions and homogenized. The fecal suspension was then centrifuged at 600 × g for 3 min at 4°C to collect the fecal bacteria suspension, followed by centrifugation at 12,000 × g for 10 min to collect the fecal bacteria. The bacteria were resuspended in fresh, pre-reduced Mega Medium at half the original suspension volume. CA was dissolved in DMSO and added to each co-culture system to achieve a final concentration of 100 μM. The cultures were incubated at 37°C under anaerobic conditions for 48 h and then stored at −80°C until further analysis. After thawing the cultures at 4°C, they were mixed with an equal volume of methanol and then sonicated at 4°C for 30 min. The samples were subsequently centrifuged at 13,000 × g for 15 min at 4°C and evaporated under nitrogen. The dried residue was dissolved in 200 µL of a methanol-water solution (1:1, v/v) and centrifuged at 10,000 × g for 1 min at 4°C. The resulting supernatant was used for analysis, as described above.

### Fecal microbiota 16S rDNA copy number measurement

The 16S rDNA copies were measured according to a previous study with some modifications.^35^ Fecal microbiota DNA was extracted using a commercial kit. The sequences of primers used were 338F (5’-ACTCCTACGGGAGGCAGCAG-3’) and 806 R (5’-GGACTACHVGGGTWTCTAAT-3’). The linear plasmid was constructed by Shanghai Sangon Biotech Company. Different concentration gradients were prepared by diluting the standard plasmid. Real-time PCR analysis was conducted on either the plasmid or sample DNA using a qPCR mix with a real-time PCR system. A standard curve was generated by plotting the logarithm of the plasmid copy number against the CT values, facilitating the quantification of fecal DNA copy numbers.

### Construction of plasmids and AAV

The constructed plasmids and viruses were obtained from WZ Biosciences (Shandong, China). The pAV-hsyn-P2A-GFP and pAV-hsyn-Nr1h4-P2A-GFP vectors were used. The accuracy of the target gene insertion was validated through sequencing of the engineered plasmid. The plasmid, pAAV-RC, and pHelper plasmid were transfected into HEK293T cells for 72 h, after which the viruses were collected and purified. The vital titers of purified AAV-Control and AAV-Nr1h4 were measured using qPCR. FXR overexpression was validated using western blotting.

### Cell experiments

SH-SY5Y cells (#100158, BNCC, Henan, China) were cultured in DMEM/F12 supplemented with 10% fetal bovine serum, 100 U/mL penicillin, and 100 µg/mL streptomycin at 37°C in a humidified atmosphere with 5% CO_2_. To study the pro-apoptotic effects of DCA, cells were harvested 24 h after incubation with DCA (25, 50, or 75 µM). In the JC-1 test, cells were treated with DCA in 12 h. To study the effect of ASBT upregulation on DCA-induced apoptosis, cells were treated with dexamethasone (DEX) (#D137736, Aladdin, Shanghai, China) for 6 h followed by DEX and DCA (25 µM) treatment for 96 h. The medium was replaced every 24 h. To investigate the effect of ASBT inhibition on DCA-induced apoptosis, cells were treated with GSK (#HY-16643, MedChemExpress, New Jersey, USA) for 6 h followed by GSK and DCA (75 µM) treatment for 24 h. For the FXR inhibition study, cells were treated with guggulsterone (Gugg) (#G276180, Aladdin, Shanghai, China) for 4 h followed by DCA (75 µM) treatment for 24 h. For the caspase 3 inhibition study, cells were treated with ZDF for 2 h followed by DCA (75 µM) for 24 h. Caspase 3 activity was tested using a commercial kit (#C1115, Beyotime, Shanghai, China). Cytotoxicity was measured using a Cell Counting Kit-8 (#AQ308, Aoqing, Beijing, China). Mitochondrial membrane potential was measured using a JC-1 assay (M8650, Solarbio, Beijing, China) and detected using a fluorescence microplate reader. Apoptosis was characterized using a TUNEL Apoptosis Assay Kit (#T2130, Solarbio, Beijing, China) and detected using a fluorescence microplate reader.

### Statistical analysis

Comparisons between groups were performed using an unpaired two-tailed t test (between two groups), one-way ANOVA followed by Dunnett’s post hoc test (for more than two groups with one variable), or two-way ANOVA (for the mice treated with ZDF and different diets) followed by Tukey’s post hoc test unless otherwise specified. Statistical analyses were performed with GraphPad Prism 9.0 (GraphPad Software, CA, USA).

## Results

### Dietary cholesterol promoted cognitive impairment in obese mice

**Figure 1.**
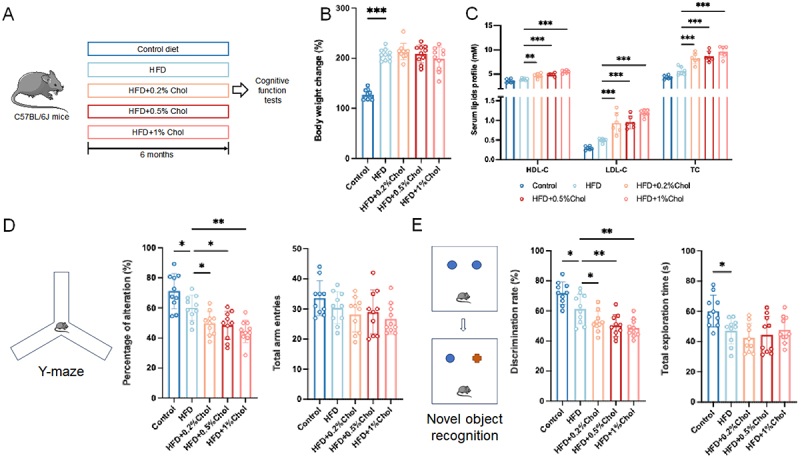
Dietary cholesterol promotes cognitive impairment in HFD-fed mice. (A) experimental framework. Mice were fed with a control, HFD, and HFD with various doses of cholesterol (0.2%, 0.5%, and 1%) for 6 months. Then, Y-maze and nor tests were performed on each mouse. (B) body weight change (*n* = 10). (C) serum lipids profile (*n* = 6). (D) percentage of alteration and total arm entries in the Y-maze test (*n* = 10). (E) discrimination rate and total exploration time in the nor test (*n* = 10). Statistical analysis was performed using a one-way ANOVA followed by Dunnett post hoc test. * *p* < 0.05, ** *p* < 0.01, *** *p* < 0.001.

A mouse model of cognitive impairment induced by an HFD was used, considering that high cholesterol intake often accompanies high fat intake. As shown in Figure 1A, mice were fed a control diet, an HFD, or an HFD with varying cholesterol concentrations (0.2%, 0.5%, or 1% cholesterol) for six months. Dietary cholesterol had no impact on body weight or food intake in obese mice (Figure 1B and Figure S1A). All doses of cholesterol significantly increased the serum lipids (HDL-C, LDL-C, and total cholesterol) in obese mice (Figure 1C). Dietary cholesterol at 0.2%, 0.5%, and 1% significantly increased plasma LDL-C in obese mice by 1.89-fold, 1.92-fold, and 2.39-fold, respectively, while total serum cholesterol levels increased to 1.19-fold, 1.24-fold, and 1.39-fold those of obese mice.

The cognitive functions of the mice were assessed using Y-maze tests, which evaluate working memory, as well as NOR tests, which assess recognition memory. In the Y-maze test, HFD reduced the percentage of alternation in mice by approximately 15.5%, while 0.2%, 0.5%, and 1% cholesterol further significantly decreased the percentage of alternation of obese mice by 17.2%, 19.5%, and 26.0%, respectively (Figure 1D). There was no significant difference in the total number of arm entries between groups, indicating that the significant differences in percentage of alternation were not due to differences in total arm entries. This suggests that dietary cholesterol impairs working memory in obese mice. However, there was no significant variation in the total number of arm entries across the groups, indicating that dietary cholesterol did not significantly affect on the motor abilities of obese mice. The lack of significant differences in total arm entries rules out the possibility that variations in percentage of alternation were due to differences in arm entries. In the NOR test, dietary cholesterol at 0.2%, 0.5%, and 1% led to significant reductions in the discrimination rate of obese mice by 14.0%, 18.2%, and 20.9%, respectively (Figure 1E). The total exploration time of obese mice was reduced compared to the control group. However, dietary cholesterol had no significant effect on the total exploration time of obese mice, indicating that the differences in discrimination rates between the HFD group and the three cholesterol groups were not significantly influenced by variations in total exploration time. These findings suggest that dietary cholesterol worsened recognition memory in obese mice without significantly affecting their motor abilities. Linear regression analysis of cholesterol intake and cognitive indicators in obese mice showed that the slopes of the regression equations for cholesterol intake concerning the percentage of alternation and discrimination rate were negative, with *p*-values less than 0.01 (Figure S1B). This indicates a significant negative correlation between cholesterol intake and cognitive function in mice. Dietary cholesterol was administered to mice fed a control diet to investigate whether it alone induces cognitive impairment. Compared to the control group, dietary cholesterol alone did not significantly affect cognitive function in mice, except that 1% cholesterol reduced the percentage of alterations in the Y-maze test (Figure S1D-E). This indicates that dietary cholesterol alone has a limited impact on cognitive function in mice. Overall, these data suggested that dietary cholesterol promoted cognitive impairment in obese mice.

**Figure 2.**
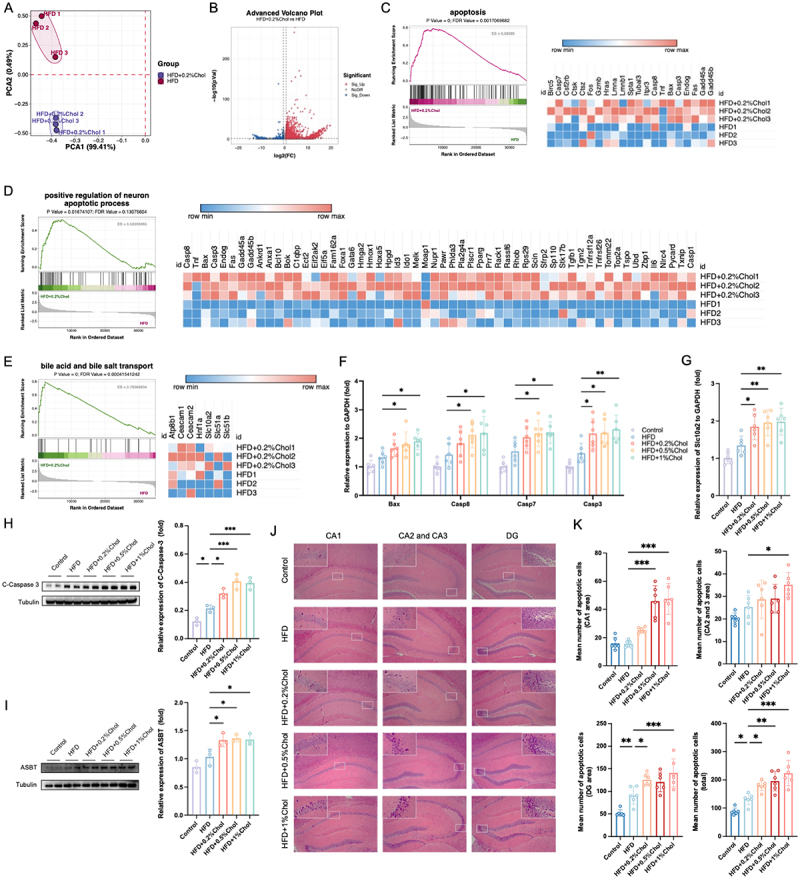
Apoptosis and bile acid transportation pathways were activated in dietary cholesterol-fed mice. (A) Principal component analysis (PCA) of mouse hippocampal transcriptome profile based on princomp function. (B) Volcano map based on DEGs (*p*_adj_ < 0.05 and Fold change ≥ 1.5 or Fold change ≤ 0.67). (C) GSEA analysis of apoptosis signaling pathway enriched in KEGG analysis (*n* = 3). (D) GSEA analysis of positive regulation of neuron apoptotic process in GO analysis (*n* = 3). (E) GSEA analysis of bile acid and bile salt transport in GO analysis (*n* = 3). (F) expression levels of genes associated with apoptosis based on qPCR tests (*n* = 6). (G) expression levels of genes associated with bile acid and bile salt transport (*Slc10a2*) (*n* = 6). (H) expression of proteins associated with apoptosis (cleaved caspase 3) based on Western blot (*n* = 3). (I) expression of proteins related to bile acid and bile salt transport (ASBT) based on Western blot (*n* = 3). (J) Representative micrographs of hippocampal tissue stained with H&E (magnification 100×). (K) the assessment of apoptotic cell numbers within the hippocampal region (*n* = 6). Statistical analysis was performed using a one-way ANOVA followed by Dunnett post hoc test. * *p* < 0.05, ** *p* < 0.01, *** *p* < 0.001.

To further understand the molecular mechanism underlying the cognitive impairment-promoting effects of dietary cholesterol, gene transcription differences in the hippocampus of mice were analyzed using transcriptomic analysis. Since the dietary cholesterol dose of 0.2% in mice is comparable to levels achievable in a human diet, the mice consuming 0.2% cholesterol were selected for subsequent omics studies, rather than the mice consuming 0.5% or 1% cholesterol. The transcriptomic profile of the HFD + 0.2%Chol group was different from that of the HFD group (Figure 2A). Compared with those in the HFD group, a total of 2013 and 245 genes in the HFD + 0.2%Chol group were up- and down-regulated, respectively (Figure 2B). The Kyoto Encyclopedia of Genes and Genomes (KEGG) pathway (apoptosis pathway) and Gene Ontology (GO) term (positive regulation of neuron apoptotic process and bile acid and bile salt transport) were significantly enriched, which was further confirmed by gene set enrichment analysis (GSEA) (Figures 2C–2E). In these pathways and terms, most of the genes were upregulated in the HFD + 0.2%Chol group. Among the main apoptotic genes, only *Casp3* was significantly upregulated in all dietary cholesterol-fed mice (Figure 2F). Additionally, the bile acid transporter gene *Slc10a2*, which encodes ASBT, also exhibited similar trends (Figure 2G). Furthermore, the protein levels of cleaved caspase 3 (C-Caspase 3) and ASBT significantly increased upon dietary cholesterol treatment (Figure 2H-I). Importantly, the three doses of cholesterol increased hippocampal C-Caspase 3 levels in obese mice by 1.46, 1.69, and 1.85 times, respectively, suggesting that high cholesterol intake substantially elevate apoptosis levels in the hippocampus. However, dietary cholesterol had no significant effect on Bax and C-Caspase 7 in the hippocampus of HFD mice (Figure S2A). The level of C-Caspase 8 in the hippocampus was significantly higher in the HFD + 1%Chol group compared to the HFD group. Neuronal apoptosis is a key factor contributing to the onset of cognitive impairment, which has been observed in individuals and animals with cognitive impairment induced by high-fat diets.^36^ H&E staining serves as a valuable tool for evaluating the number of apoptotic neurons in the hippocampus.^37^ Differences in hippocampal apoptotic cells were further confirmed by H&E staining. HFD primarily increases the number of apoptotic cells in the DG region of the mouse hippocampus (Figure 2J-K). In comparison to HFD mice, 0.2% and 0.5% cholesterol mainly induce apoptosis in the DG and CA1 regions, respectively, while 1% cholesterol increases the number of apoptotic cells in the CA1–3 and DG regions. Overall, the number of apoptotic cells in the hippocampus was significantly greater in the HFD group compared to the control group, with dietary cholesterol further exacerbating this increase in cell apoptosis. To identify the main types of apoptotic cells in the hippocampus of mice, we examined markers for neurons, microglia, and astrocytes, specifically NeuN, IBA-1, and GFAP, respectively. The Western blot and immunofluorescence results showed that, compared to the Control group, NeuN was significantly reduced in the hippocampus of HFD mice, while both IBA-1 and GFAP were significantly increased (Figure S2B-C). Furthermore, all three doses of dietary cholesterol significantly decreased NeuN levels in the hippocampus of obese mice. Compared to the HFD group, only the HFD + 0.5%Chol group showed a significant increase in IBA-1 in the hippocampus. These findings suggest that dietary cholesterol significantly reduces the number of neurons in the hippocampus of obese mice, likely contributing to neuronal apoptosis. Given the neurotoxicity of cholesterol accumulation,^6^ the cholesterol level in the hippocampus was determined to identify the potential mechanism of neuronal apoptosis. Hippocampal total cholesterol did not change significantly (Figure S2D). Additionally, the hippocampal gene expressions of *Cyp46a1*, an enzyme responsible for cholesterol clearance, did not significantly differ across the mice in each group (Figure S2E). These results suggested that dietary cholesterol does not induce cholesterol accumulation in hippocampus and other metabolites may promote the apoptosis of neurons, further exacerbating cognitive impairment in the mice.

## Dietary cholesterol increased fecal microbiota containing 7ɑ-dehydroxylase and hippocampal DCA in obese mice

**Figure 3.**
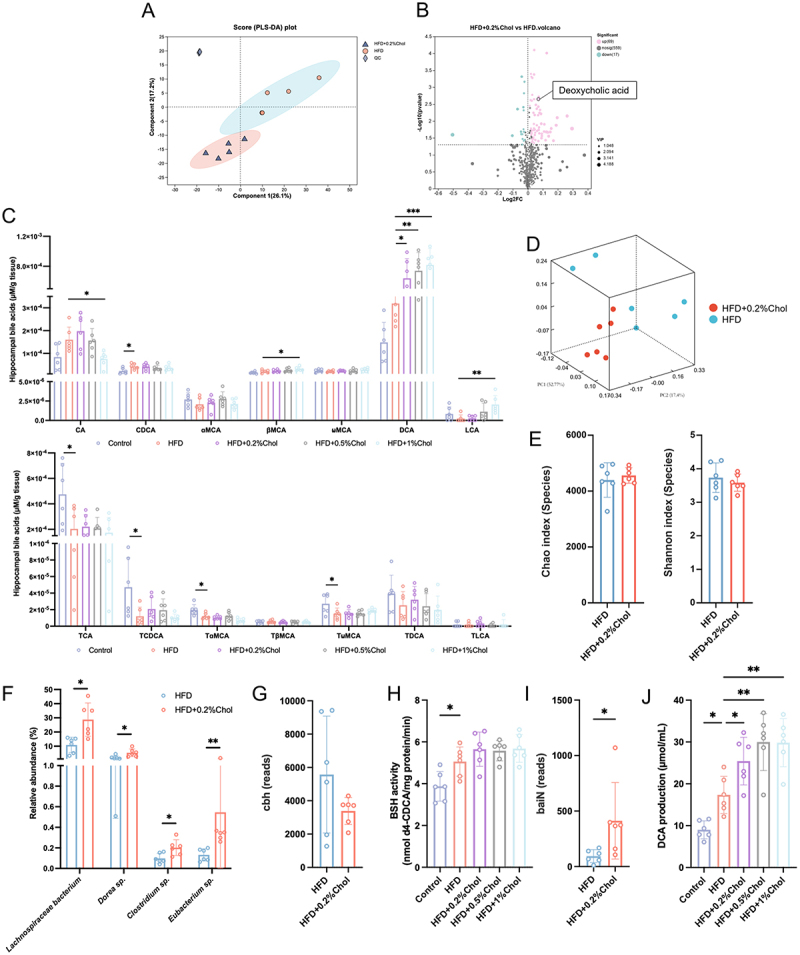
Dietary cholesterol increased fecal microbiota containing 7ɑ-dehydroxylase and hippocampal DCA in HFD-fed mice. (A) PLS-DA analysis of metabolic profile in the HFD and HFD + 0.2%Chol groups (*n* = 5). (B) volcano plots illustrate variations in metabolite levels in murine serum (*n* = 5). (C) hippocampal levels of unconjugated and taurine-conjugated BAs (*n* = 5). (D) PCoA of fecal microbiota profile in the HFD and HFD + 0.2%Chol groups at the species level (*n* = 6). (E) ɑ-diversity of fecal microbiota in the HFD and HFD + 0.2%Chol groups at the species level (*n* = 6). (F) relative abundance of *lachnospiraceae bacterium*, *Dorea* sp., *Clostridium* sp., and *Eubacterium* sp. in murine feces (*n* = 6). (G) reads number of *cbh* in fecal microbiota (*n* = 6). (H) fecal BSH activity in mice (*n* = 6). (I) reads number of *baiN* in fecal microbiota (*n* = 6). (J) *in vitro* assessment of DCA production by fecal bacteria (*n* = 6). For the differences of microbiota, the Wilcoxon rank-sum test (two-tailed test) was employed. For the other data, an unpaired t-test or one-way ANOVA followed by Dunnett post hoc test was used. * *p* < 0.05, ** *p* < 0.01, *** *p* < 0.001.

The serum metabolome was analyzed to identify the potential metabolites that mediate the cognitive impairment-promoting effects of dietary cholesterol. In the PLS-DS plot, the metabolites in the HFD and HFD + 0.2%Chol groups were separated, which is an indication that dietary cholesterol-induced significant alterations in metabolic components (Figure 3A). KEGG pathway enrichment analysis suggested that the bile secretion and primary bile acid biosynthesis pathways were enriched (Figure S3A). The serum DCA was greater in the HFD + 0.2%Chol group than in the HFD group (Figure 3B). The elevation of secondary BAs in the serum and brain has been observed in patients with cognitive impairment.^16,17^ In our study, the hippocampal BA composition was analyzed. Increased levels of CDCA, along with decreased levels of taurocholic acid (TCA), tauro-ɑ-muricholic acid (TαMCA), and TCDCA, were observed in the obese mice (Figure 3C). However, in all cholesterol-fed groups, only the levels of DCA were elevated in obese mice. Compared to the HFD group, hippocampal DCA levels in the HFD + 0.2%, 0.5%, and 1% cholesterol groups increased by 2.00, 2.32, and 2.56 times, respectively. Moreover, 1% cholesterol decreased CA and TɑMCA but increased β-muricholic acid (βMCA) and lithocholic acid (LCA) in obese mice. The BAs composition in serum was also characterized (Figure S3B). The results showed that 1% cholesterol reduced the serum levels of CA and CDCA in HFD mice, while increasing the level of LCA. Furthermore, 0.5% cholesterol only significantly elevated the serum CDCA levels. However, although there was an upward trend in the serum DCA levels of cholesterol-fed HFD mice compared to the HFD group, the difference was not statistically significant. Interestingly, the hippocampal DCA levels in cholesterol-fed HFD mice were significantly higher than in the HFD mice, which may be related to the elevated expression of the DCA transporter ASBT in the hippocampus of cholesterol-fed HFD mice, and this will be explored further in subsequent studies. BAs, including DCA, CDCA, and LCA, act as ligands for FXR and further modulate several biological processes. Increases in FXR expression were observed in dietary cholesterol-consuming mice (Figure S3C). The effects of dietary cholesterol alone on hippocampal DCA levels in mice fed a control diet were also investigated. The results showed that only 1% cholesterol significantly increased hippocampal DCA levels by 3.96 times, while 0.2% and 0.5% cholesterol had no significant effect on DCA levels (Figure S3D). This may explain why dietary cholesterol alone has a limited impact on cognitive function in mice.

BAs are synthesized from cholesterol in the liver and are excreted into the intestine after conjugation with either taurine or glycine. They can be deconjugated by BSH present in the intestinal microbiota, and the resulting deconjugated primary BAs can be further converted into secondary BAs by bacterial 7ɑ-dehydroxylase.^38^ The mRNA and protein expressions of the hepatic BA synthesis enzymes CYP7a1, CYP8b1, and CYP7b1 were significantly reduced under HFD feeding, but dietary cholesterol did not further notably affect these mRNA levels (Figure S3E-F). This suggests that the BA changes induced by dietary cholesterol in obese mice may be mediated by the gut microbiota rather than hepatic BA synthesis.

The gut microbiota profiles in mice from the HFD group and the HFD + 0.2%Chol group were analyzed via metagenomic sequencing. PCoA revealed differences in the fecal microbiota profiles at the species level (Figure 3D) and genus level (Figure S4A). The Chao index and Shannon index did not significantly differ between the fecal microbiota of these two groups (Figure 3E and Figure S4B), indicating that 0.2% cholesterol did not significantly alter the alpha diversity of the intestinal microbiome in obese mice. At the phylum level, a notable increase in *Firmicutes* and a decreasing trend of *Actinobacteria* exhibited HFD + 0.2%Chol group (Figure S4C-D). At the genus level, an increase in *Dorea* and a genus belonging to the *Lachnospiraceae* family was observed in the HFD + 0.2%Chol group, while a genus belonging to *Erysipelotrichaceae* family and another from the *Atopobiaceae* family exhibited greater abundance in the HFD group (Figure S5A-B). Notably, at the species level, *Clostridium* sp. that were abundant in BSH and 7ɑ-dehydroxylases showed a significant increase in the HFD + 0.2%Chol group relative to their abundance in the HFD group (Figure 3F and Figure S5C-D). The presence of *Eubacterium* sp. producing BSH and 7ɑ-dehydroxylases-enriched *Lachnospiraceae bacterium*, *Lachnospiraceae bacterium* 10–1 and *Dorea* sp. also elevated in the HFD + 0.2%Chol group (Figure 3F). The reads of genes encoding BSH and 7ɑ-dehydroxylase were further analyzed. No substantial variation was observed in the quantity of *cbh* (coding for BSH) reads between the two groups (Figure 3G). Similarly, in Figure 3H, analysis of BSH activity in mouse feces revealed that high cholesterol did not significantly alter fecal BSH activity in the obese mice. However, *baiN*, which encodes one of the enzymes involved in the dehydroxylation of primary BAs,^39^ was significantly enriched in the HFD + 0.2%Chol group (Figure 3I). Furthermore, *in vitro* co-culturing fecal microbiota with CA revealed that higher intake of cholesterol enhanced the fecal microbiota’s capacity to produce DCA from CA in HFD mice (Figure 3J). These findings suggested that dietary cholesterol increases the abundance of microbes involved in secondary BA synthesis, consistent with the observed elevated DCA levels in dietary cholesterol-treated mice.

The gut microbiota represents a complex ecosystem, where interactions among its members influence the host health. Using the iNAP tool, a network analysis was performed at the species level for two groups of microbes. The results showed that the network of the HFD group consisted of 48 nodes and 172 edges, while the HFD + 0.2%Chol group had 46 nodes and 161 edges, indicating that more members in the HFD group were involved in key interactions (Figure S6A). Additionally, the proportion of negative correlations in the network of the HFD and HFD + 0.2%Chol groups was 32% and 49.7%, respectively. As is shown in Figure S6B, there were more negative correlations between microbes in the HFD + 0.2%Chol group compared to the HFD group, suggesting that dietary cholesterol drives the gut microbiota toward a competitive rather than cooperative relationship.

In the co-occurrence network, the degree of a node reflects importance within the network, as it represents direct connections to other nodes. The weighted degree of each node was calculated to represent its relative importance within the network (Figure S6C). In the HFD group, *Lachnospiraceae* bacterium and *Dorea* sp. ranked 2nd and 12th, respectively, in terms of weighted degree among all nodes. However, in the HFD + 0.2%Chol group, they ranked 1st and 3rd, respectively, indicating that under the influence of cholesterol, the importance of *Lachnospiraceae* bacterium and *Dorea* sp. increased, possibly playing a leading role in the microbial network.

## Dietary cholesterol-modulated gut microbiota drove HFD-induced cognitive impairment

**Figure 4.**
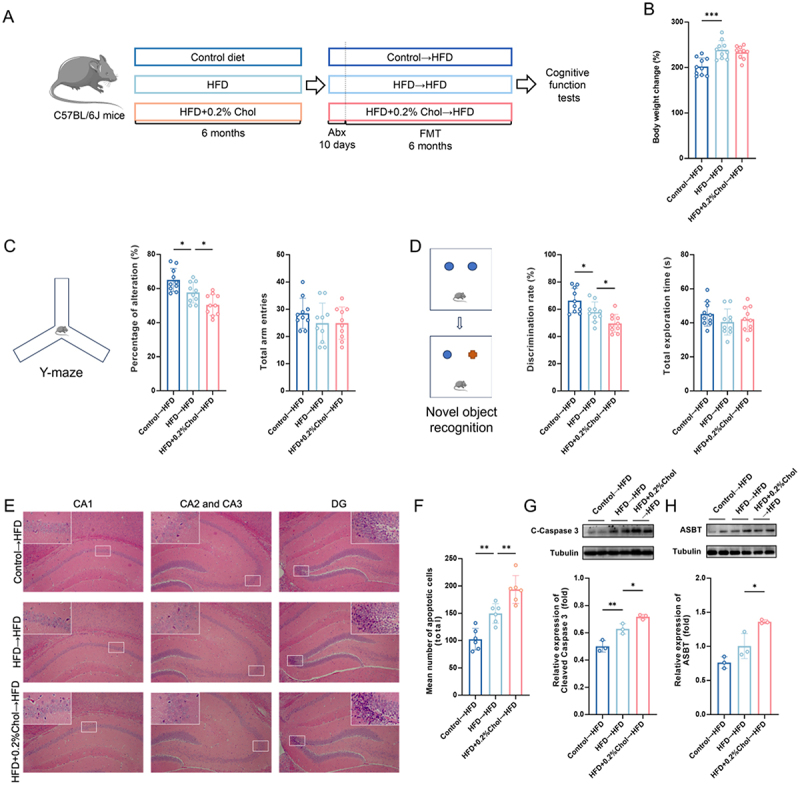
Pro-cognitive impairment effects of dietary cholesterol can be transferred by gut microbiota. (A) experimental framework. (B) body weight change (*n* = 10). (C) percentage of alteration and total arm entries in the Y-maze test (*n* = 10). (D) discrimination rate and total exploration time in the nor test (*n* = 10). (E) Representative micrographs of hippocampal tissue stained with H&E (magnification 100×). (F) the assessment of apoptotic cell numbers within the hippocampal region (*n* = 6). (G) protein level of C-Caspase 3 (*n* = 3). (H) protein expression of ASBT (*n* = 3). Statistical analysis was performed using one-way ANOVA followed by Dunnett post hoc test. * *p* < 0.05, ** *p* < 0.01, *** *p* < 0.001.

The fecal microbiota profiles exhibited significant alterations in the HFD + 0.2%Chol group. Consequently, we performed FMT to elucidate the role of the microbiota in the cognitive impairment-promoting effects of dietary cholesterol. After the administration of an antibiotic cocktail (Abx), the levels of fecal microbiota in the mice were significantly reduced, indicating the successful establishment of Abx-induced pseudo-germ-free mice (Figure S7A). As illustrated in Figure 4A, fecal microbiota from the control diet, HFD, and HFD + 0.2%Chol diet-fed mice were transplanted to three groups of mice fed an HFD for six months. After 7 days of FMT, the fecal microbiota in all three groups of mice significantly increased, indicating successful colonization of the transplanted fecal microbiota (Figure S7B). Mice receiving fecal samples from the HFD and HFD + 0.2%Chol groups had significantly greater body weights than those receiving samples from the control group (Figure 4B). The food intake of mice in the three groups exhibited no significant differences (Figure S8A).

The Y-maze test revealed that the working memory abilities of the mice in the Control→HFD group were greater than those in the HFD→HFD group (Figure 4C). Conversely, mice in the HFD + 0.2%Chol→HFD group exhibited poorer working memory than did those in the HFD→HFD group. Similarly, compared to the fecal microbiota from the HFD group, those from the HFD + 0.2%Chol group exhibited significant impairment in recognition memory (Figure 4D). H&E staining and C-Caspase 3 protein analysis both indicated that mice receiving fecal microbiota from the HFD + 0.2%Chol group exhibited a higher number of apoptotic cells (Figure 4E-G). This was particularly evident in the hippocampal CA1 and DG areas, as observed through H&E staining, compared to mice receiving fecal microbiota from the HFD group (Figure S8B). These results indicated that dietary cholesterol-modulated gut microbiota drives HFD-induced cognitive impairment.

The relative abundance of 7α-dehydroxylase-containing bacteria in mouse fecal microbiota after FMT was characterized by measuring the fecal microbiota’s capacity to produce DCA from CA. The DCA production by fecal microbiota in the HFD + 0.2%Chol→HFD group exceeded that observed in the HFD→HFD group, indicating a greater presence of 7α-dehydroxylase-containing bacteria in the HFD + 0.2%Chol→HFD group (Figure S8C).

Mice receiving fecal samples from the HFD + 0.2%Chol group exhibited higher levels of ASBT and FXR compared to those receiving samples from the HFD group (Figure 4H and Figure S8D). This suggested that more BAs may be transported into the hippocampal tissue of the HFD + 0.2%Chol→HFD group, which is associated with neuronal apoptosis.

To further confirm the causal role of gut microbiota in dietary cholesterol-induced cognitive impairment, a reverse FMT experiment was conducted. Fecal microbiota from control diet-fed mice, HFD-fed mice, and HFD + 0.2% cholesterol-fed mice were transplanted into recipient mice maintained on an HFD + 0.2% cholesterol diet. Notably, compared to mice receiving microbiota from the HFD + 0.2% cholesterol group, those transplanted with microbiota from either control or HFD-fed mice exhibited significant mitigation of cognitive impairment and reduced neuronal apoptosis (Figure S9). These findings further support the critical involvement of gut microbiota in mediating the detrimental effects of dietary cholesterol on cognitive function in obese mice.

## DCA induced cognitive impairment in obese mice

**Figure 5.**
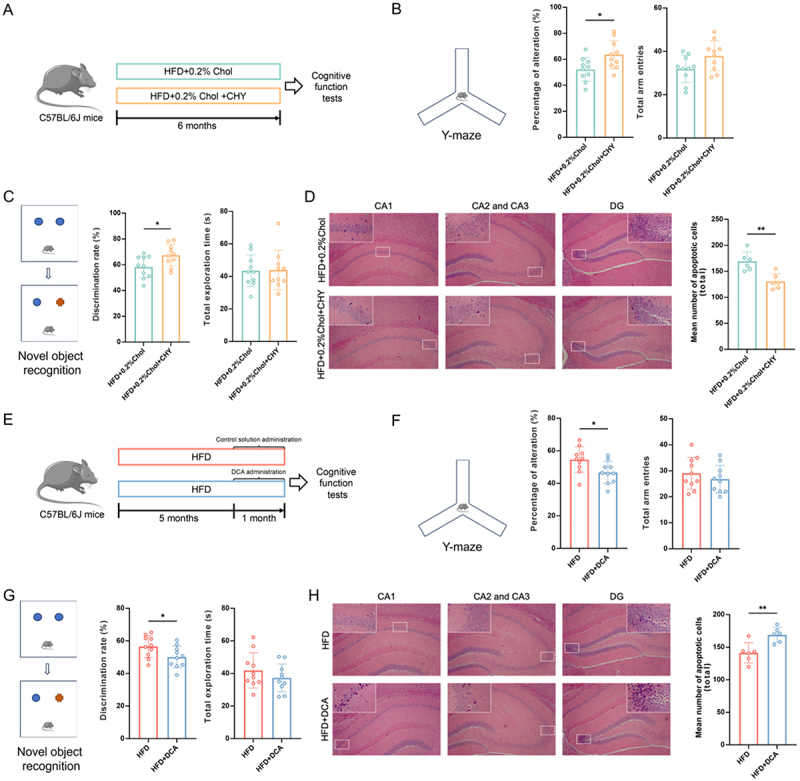
Deoxycholic acid-induced cognitive impairment in HFD-fed mice. (A) experimental framework. (B) percentage of alteration and total arm entries in the Y-maze test (*n* = 10). (C) discrimination rate and total exploration time in the nor test (*n* = 10). (D) Representative micrographs of hippocampal tissue stained with H&E (magnification 100×) and the assessment of apoptotic cell numbers within the hippocampal region (*n* = 6). (E) experimental framework. (F) percentage of alteration and total arm entries in the Y-maze test (*n* = 10). (G) discrimination rate and total exploration time in the nor test (*n* = 10). (H) Representative micrographs of hippocampal tissue stained with H&E (magnification 100×) and the assessment of apoptotic cell numbers within the hippocampal region (*n* = 6). Statistical analysis was performed using an unpaired t-test. * *p* < 0.05, ** *p* < 0.01, *** *p* < 0.001.

To determine the role of BAs in dietary cholesterol-induced cognitive impairment, a bile acid sequestrant, CHY, was administered orally to HFD + 0.2%Chol-fed mice for six months (Figure 5A). CHY reduced body weight gain in HFD + 0.2%Chol-fed mice but had no obvious effect on food intake (Figure S10A-B). Moreover, CHY significantly increased total BAs in the feces while significantly decreasing total BAs in the serum and brain (Figure S10C). Notably, the Y-maze test revealed that CHY significantly improved the percentage of alternation in HFD + 0.2%Chol diet-fed mice (Figure 5B). In the NOR test, CHY-treated mice also exhibited a greater discrimination rate (Figure 5C). These results indicated that bile acid sequestration significantly improved working memory and recognition memory in HFD + 0.2%Chol diet-fed mice, suggesting the involvement of bile acid accumulation in the cognitive decline promoted by dietary cholesterol. CHY reduced neuronal apoptosis in the hippocampus of HFD + 0.2%Chol mice, especially in the CA1 and DG regions (Figure 5D and Figure S10D-E).

Dietary cholesterol induced higher hippocampal levels of DCA in the obese mice. To investigate whether DCA mediates the cognitive impairment-promoting effects of dietary cholesterol in HFD mice, DCA was infused into the hippocampal tissue of HFD-fed mice for one month (Figure 5E). The administration of DCA exhibited no obvious effect on body weight or food intake (Figure S10F-G). In the Y-maze test, DCA significantly reduced the spontaneous alternation in obese mice, indicating that DCA impaired working memory (Figure 5F). Similarly, the results of the NOR test suggested that DCA impaired the recognition memory of obese mice (Figure 5G). Compared to the HFD group, the HFD+DCA group showed a significant increase in hippocampal neuronal apoptosis, particularly in the DG region. (Figure 5H and Figure S10H-I). These results suggested that DCA induces cognitive impairment in obese mice, which means dietary cholesterol can promote cognitive decline by increasing gut microbiota-derived DCA.

## Hippocampal ASBT regulate DCA-induced cognitive impairment in obese mice

**Figure 6.**
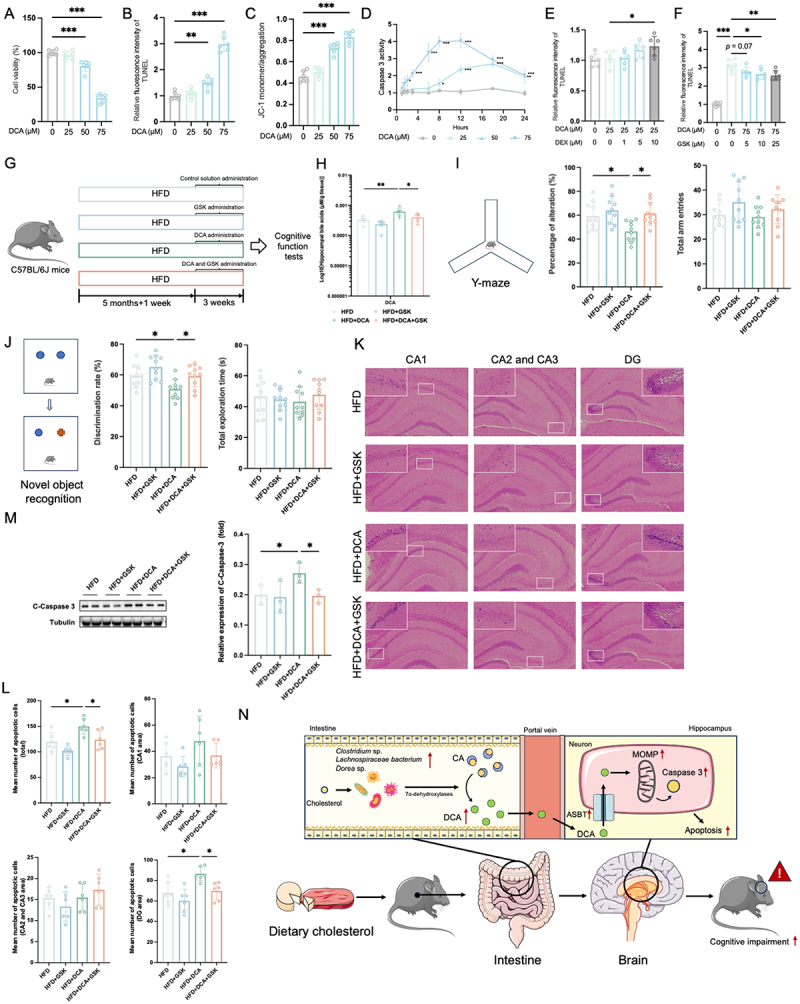
ASBT regulates DCA-induced neuronal apoptosis. (A) cell viability after 25–75 μM DCA treatments in SH-SY5Y for 24 h (*n* = 6). (B) TUNEL density after 25–75 μM DCA treatments in SH-SY5Y for 24 h (*n* = 6). (C) JC-1 monomer/aggregation after 25–75 μM DCA treatments in SH-SY5Y for 12 h (*n* = 6). (D) dynamics of caspase 3 activity after 25–75 μM DCA treatments in SH-SY5Y for 24 h (*n* = 6). (E) TUNEL density after DEX (1–10 μM) and DCA (25 μM) co-treatments in SH-SY5Y for 96 h (*n* = 6). (F) TUNEL density after GSK (5–25 μM) and DCA (75 μM) co-treatments in SH-SY5Y for 24 h (*n* = 6). (G) experimental design. (H) effects of GSK on hippocampal DCA level (*n* = 6). (I) percentage of alteration and total arm entries in the Y-maze test (*n* = 10). (J) discrimination rate and total exploration time in the novel object recognition test (*n* = 10). (K) Representative images of hippocampus H&E staining (magnification 100×) and the quantification of apoptotic cells in the hippocampus (*n* = 6). (L) effects of DCA on the number of apoptotic cells in different regions of the mouse hippocampus (*n* = 6). (M) protein expression of cleaved-caspase-3 (*n* = 3). (N) schematic diagram of the mechanism of dietary cholesterol promotes HFD-induced cognitive impairment. In (a-c) and (e-f), statistical analysis was performed using one-way ANOVA followed by Dunnett post hoc test. In (D) and (H-L), statistical analysis was performed using two-way ANOVA followed by Tukey post hoc test. * *p* < 0.05, ** *p* < 0.01, *** *p* < 0.001. DEX, dexamethasone; GSK, GSK2330672.

DCA induces apoptosis in several cells.^40^ The role of DCA in neuronal apoptosis was tested in a cell model. DCA at concentrations of 50–75 µM was cytotoxic to SH-SY5Y cells, and the increase in TUNEL intensity indicated the pro-apoptotic effects of DCA on SH-SY5Y cells (Figures 6A–6B). Mitochondrial outer membrane permeabilization (MOMP)-caspase 3 may involve in the pro-apoptosis of BAs.^40^ After 50 and 75 DCA µM treatment, mitochondrial membrane potential decreased, indicating the DCA-induced MOMP (Figure 6C). A time course experiment indicated the caspase 3 activity in cells was sequentially elevated by 50 and 75 µM DCA (Figure 6D). These results demonstrated the pro-apoptotic effects of DCA on neuronal cells in a dose-dependent manner. To test whether other hydrophobic bile acids, such as LCA and CDCA, might also exhibit neurotoxicity, we evaluated the toxicity of LCA and CDCA on SH-SY5Y cells. The results indicate that, at the same concentration, both LCA and CDCA exhibit neurotoxicity (Figure S11A-B). However, according to our measurements, the concentrations of LCA and CDCA in the hippocampus of mice are approximately 0.00075–0.0056 times and 0.045–0.15 times that of DCA, respectively. Therefore, we tested the neurotoxicity of LCA at concentrations ranging from 0.001 to 0.005 times the high-toxic concentration of DCA (75 μM), and CDCA at concentrations ranging from 0.045 to 0.15 times that of DCA. The results showed that these concentrations of LCA and CDCA were not toxic to neuronal cells (Figure S11C-D). Additionally, we tested whether these concentrations of LCA and CDCA could synergistically enhance the cytotoxicity of DCA. The results indicated no significant increase in the cytotoxicity of DCA when combined with these concentrations of LCA and CDCA (Figure S11E-F). This finding highlights the unique neurotoxicity of DCA compared to other bile acids in this study.

ASBT actively transports BAs into cells, and its high expression mediates the accumulation of BAs.^26^ We manipulated neuronal ASBT expression using an activator, DEX, and an inhibitor, GSK to investigate the causal role of hippocampal DCA accumulation in neuronal apoptosis.^26^ The CCK-8 assay results showed that 0.5–10 μM DEX and 1–25 μM GSK did not exhibit significant cytotoxicity on SH-SY5Y cells (Figure S12A-B). Under DEX stimulation, DCA can induce neuronal apoptosis at concentrations as low as 25 µM (Figure 6E). However, the pro-apoptotic effect of 75 µM DCA was significantly attenuated by the stimulation of GSK (Figure 6F). This suggests that ASBT may play a role in the pro-apoptotic effects of DCA. Upregulation of ASBT facilitates the cellular uptake of DCA, thereby enhancing its pro-apoptotic activity. Conversely, the downregulation of ASBT reduces cellular uptake of DCA, thereby attenuating its pro-apoptotic effects.

To elucidate whether ASBT has regulatory effect on DCA-induced neuronal apoptosis and murine cognitive impairment, hippocampal ASBT was inhibited using GSK (Figure 6G). As shown in Figure 6H, DCA infusion into the hippocampus of mice significantly increased the hippocampal DCA levels in obese mice. However, compared to the HFD+DCA group, the HFD+GSK+DCA group exhibited a significant reduction of approximately 36.1% in hippocampal DCA levels, demonstrating that *in vivo* inhibition of ASBT significantly reduced neuronal DCA accumulation. Compared to the HFD group, DCA significantly decreased the percentage of alteration and discrimination rate in mice, while GSK notably improved the cognitive deficits induced by DCA (Figures 6I–6J). Similarly, DCA significantly increased the number of apoptotic neurons and the expression of C-Caspase 3 in the hippocampus of HFD mice (Figures 6K–6M). However, this effect was markedly inhibited with GSK treatment. These results suggest that ASBT-mediated DCA accumulation in neuronal cells contributes to neuronal apoptosis and cognitive impairments in mice.

## Inhibition of caspase 3 improves the cognitive impairment-promoting effects of dietary cholesterol

The accumulation of DCA in neuronal cells can induce apoptosis, yet the molecular mechanisms remain unclear. FXR plays a role in the progression of neurological decline associated with multiple liver diseases, and knocking out FXR can protect against neuronal apoptosis in ischemic injury in mice.^25,41,42^ Additionally, the FXR agonists CDCA and GW4064 can induce cardiomyocyte apoptosis by activating FXR, leading to mitochondrial permeability transition and caspase 3 activation.^43^ We speculated that DCA may induce neuronal apoptosis by activating FXR, leading to caspase 3 activation and subsequently causing cell apoptosis. To elucidate the role of FXR and caspase 3 in neuronal apoptosis, the FXR antagonist Gugg or caspase 3 inhibitor ZDF) was administered to SH-SY5Y cells. Gugg significantly reduced DCA-induced cell apoptosis and caspase 3 activity (Figure S12C-D). Moreover, DCA-induced neuronal apoptosis was significantly inhibited by ZDF (Figure S12E). These data suggested that DCA may induces neuronal apoptosis at least partly through FXR/caspase 3.

To elucidate the specific role of hippocampal FXR in response to dietary cholesterol, hippocampal FXR was overexpressed in HFD- or HFD + 0.2%Chol-treated mice (Figure S13A). AAV was administered to target hippocampal neurons for the overexpression of FXR after four months of feeding, which was followed by an additional two months of feeding. In contrast to those in the AAV-Control-treated group, the hippocampal FXR protein levels in the HFD-fed and HFD + 0.2%Chol-fed mice were significantly greater in the AAV-*Nr1h4*-treated group (Figure S13B). FXR overexpression did not change body weight or food intake in HFD-fed or HFD + 0.2%Chol-fed mice (Figure S13C). In the Y-maze test, overexpression of FXR resulted in a significant decrease in spontaneous alternation in HFD + 0.2%Chol-fed mice (Figure S13D). However, in the NOR test, FXR overexpression did not significantly impact the cognitive function of the mice (Figure S13E). Notably, FXR overexpression did not significantly increase the number of apoptotic cells and C-Caspase 3 in HFD-fed and HFD + 0.2%Chol-fed mice, although upward trends were observed in the FXR overexpressed groups (Figure S13F-H). The results indicated that the overexpression of FXR has a limited impact on cognitive function in mice and does not significantly affect hippocampal cell apoptosis, suggesting that FXR may not be the main factor mediating the cognitive impairment induced by dietary cholesterol.

To elucidate the role of caspase 3 in the cognitive impairment-promoting of dietary cholesterol, mice were pretreated with an HFD or HFD + 0.2%Chol for five months and then infused with ZDF or control solution for one month (Figure S14A). The differences in body weight change and food intake were not significant (Figure S14B). ZDF improved HFD- or HFD + 0.2%Chol-induced cognitive decline (Figure S14C-D). Importantly, no significant differences were observed in cognitive function between the HFD+ZDF and HFD + 0.2%Chol+ZDF groups. Inhibition of caspase 3 significantly reduced the number of apoptotic cells in the DG region of the hippocampus in obese mice, as well as the number of apoptotic neurons in the CA1–3 and DG regions of the hippocampus in HFD + 0.2%Chol-fed mice (Figure S14E-F). Correspondingly, the activation of caspase 3 was also reduced in these two ZDF-treated groups (Figure S14G). Taken together, these results indicated that dietary cholesterol promotes HFD-induced cognitive impairment in a caspase 3 activation-dependent manner.

## Discussion

The dietary cholesterol intake among Americans is approximately 137 mg/kcal, with 42% contributed by sources such as poultry, red meat, and processed meat and 25% contributed by eggs.^44^ According to data from the China Health and Nutrition Survey, 36.9% of the population had a daily dietary cholesterol intake of more than 300 mg, while 12.6% had a daily intake exceeding 500 mg.^45^ Although the risk of dietary cholesterol is considered to be less significant than previously thought, the health risks associated with high intake remain considerable.^3^ In recent years, the connection linking dietary cholesterol to cognitive function has increasingly been reported.^10^ It is believed that the gut microbiota and its metabolites could play a role in the development of cognitive impairment.^46^ However, the understanding of the relationships among dietary cholesterol, the gut microbiota and its metabolites, and cognitive impairment remains limited, greatly hindering the identification of microbial and metabolite targets to improve diet-related cognitive impairment.

Here, we demonstrate that long-term intake of dietary cholesterol promotes HFD-induced cognitive impairment. Dietary cholesterol elevated the serum and hippocampal levels of DCA in obese mice by increasing the gut microbiota containing 7α-dehydroxylase, including *Lachnospiraceae* bacterium, *Dorea* sp., and *Clostridium* sp. Elevated levels of DCA enter hippocampal neurons via ASBT, acting on mitochondria and subsequently activating caspase 3. This contributes to neuronal apoptosis, which further promotes cognitive impairment in HFD mice (Figure 6N).

Following dietary cholesterol intake, a portion is absorbed through the small intestine into the bloodstream, while the remainder enters the large intestine. Within the liver, absorbed cholesterol undergoes enzymatic transformations involving key enzymes, including CYP7A1, CYP27A1, and CYP8B1, ultimately leading to the synthesis of the primary BAs CA and CDCA.^38^ In mice, CDCA undergoes further biotransformation into muricholic acid. After conjugation with taurine or glycine, these conjugated primary bile acids are stored in the gallbladder. Upon ingestion, BAs are released into the intestine to aid in the absorption of lipids. Simultaneously, BAs are deconjugated by intestinal microbiota containing BSH and metabolized by 7ɑ-dehydroxylase-containing intestinal microbiota into secondary BAs, mainly DCA and LCA.^38,47^ BAs that are reabsorbed in the intestine can reach various organs via the bloodstream, where they exert their potential physiological regulatory effects. Previous studies have reported that an HFD leads to increased serum DCA levels in mice, which promotes the development of hepatocellular carcinoma.^48^ Consuming a Western-style diet leads to an increase in *Clostridium*-mediated DCA, which activates FXR and subsequently causes a deficiency in Paneth cells.^49^ However, the regulation of bile acids by dietary cholesterol is complex, with different studies reporting varying results. Administering an HFD diet containing 0.2% cholesterol to C57BL/6 mice for 14 months led to a marked elevation in serum levels of TCDCA, GCA, TCA, TUDCA, tauro-hyodeoxycholic acid, and TDCA compared to those in mice fed an HFD.^15^ Furthermore, in comparison to mice fed an HFD, mice fed an HFD containing 2% cholesterol for 24 weeks exhibited significant increases in the liver levels of DCA and α/βMCA, while the levels of UDCA, taurine-conjugated α/βMCA, TUDCA, and TCDCA significantly decreased.^14^ The differences in these results may be attributed to variations in the complex cholesterol and bile acid homeostatic mechanisms in the body, as well as differences in the response of gut microbiota to cholesterol and BAs. Cholesterol metabolism enzyme activity is influenced by the amount and duration of cholesterol intake, resulting in varying levels of cholesterol-to-BA conversion.^50,51^ Furthermore, the negative feedback regulatory mechanism of BAs leads to differences in the synthesis levels of primary BAs.^52^ Additionally, the gut microbiota and its interaction with different BAs may also contribute to variations in BA metabolism, thereby generating different types of conjugated and secondary BAs.^53^

There has been a growing focus on the impact of gut microbiota and its metabolites in regulating cognitive function. In our study, the abundances of the phylum *Firmicutes*, a genus belonging to the family *Lachnospiraceae*, the genus *Dorea*, the species *Lachnospiraceae bacterium*, *Lachnospiraceae bacterium* 10–1, *Dorea* sp., *Eubacterium* sp., and *Clostridium* sp. were increased by dietary cholesterol feeding. An HFD typically elevates the abundance of *Firmicutes*, and this change has been associated with obesity and the subsequent chronic diseases, including cognitive impairment.^54^ An increase in the abundance of *Lachnospiraceae* has also been observed in an AD mouse model.^55^ Members of *Lachnospiraceae* contain enzymes involved in 7α-dehydroxylation, converting primary BAs into secondary BAs, including DCA and LCA.^56^ The abundance of *Dorea* was found to be greater in AD patients and was negatively associated with cognitive test scores, suggesting that *Dorea* may contribute to the pathogenesis of AD.^57^ Bacteria belonging to this genus were found to possess the ability to produce secondary BAs.^58^ A comparison between Egyptian AD patients and healthy volunteers revealed a greater abundance of *Clostridium* cluster IV and a lower abundance of *Actinobacteria* in AD patients.^59^ A study based on Chinese AD patients indicated an increased abundance of *Clostridium* (clusters IV, XIVa, and XVIII) and *Eubacterium* in AD patients.^60^ Notably, *Clostridium* contains BSH capable of deconjugating BAs, while both *Clostridium* and *Eubacterium* contain 7α-dehydroxylase capable of producing secondary BAs.^38^ This may explain the observed increase in DCA in the obese mice, which was induced by dietary cholesterol in this study. In agreement with our results, *Clostridium* was more enriched in mice consuming a diet high in fat and cholesterol compared to those on an HFD alone.^15^

The presence of BA transporters and receptors in the brain suggests that BAs may play a role in regulating cognitive function.^46^ In our study, dietary cholesterol upregulated the expression of the BA transporter ASBT in the hippocampus, which may mediate the accumulation of DCA in the hippocampus. CHY is an intestinal BA sequestrant that reduces the absorption of BAs in the intestine, consequently decreasing circulating BAs in the body. CHY improved cognitive impairment in dietary cholesterol-fed mice, suggesting a pathogenic role of bile acid accumulation in cognitive impairment in mice. Furthermore, intrahippocampal administration of DCA into the obese mice exacerbated cognitive impairment. This established a cause-and-effect relationship between DCA and cognitive impairment, identifying DCA as a mediator of this impairment.

Neuronal apoptosis is a vital pathogenic factor in cognitive impairment. Under various stimuli, caspase 3 is cleaved and activated, which further cleaves hundreds of different proteins, inducing apoptosis. Our research indicated that DCA at concentrations of 50–75 µM lead to caspase-3 cleavage and subsequent neuronal cell apoptosis. Upregulation of ASBT expression caused neuronal apoptosis with only 25 µM DCA. Conversely, downregulating ASBT expression reduced neuronal cell apoptosis induced by 75 µM DCA. In the obese mice, the administration of GSK, an ASBT inhibitor, significantly improved DCA-induced neuronal apoptosis and cognitive impairment. Effects of ASBT-mediated BA accumulation on neurological function has been reported in animal studies. Ren et al. elevated intestinal ASBT levels by injecting DEX, which significantly increased the levels of conjugated primary BAs in the serum and brain of rats, exacerbating cognitive impairment in aged rats.^23^ However, GSK was able to counteract the effects of DEX. Similar results were also observed in mice. Xie et al. observed elevated ASBT levels and increased BA levels in the brains of mice with liver failure-induced neurological impairment.^61^ Administration of an ASBT inhibitor (SC-435) to these mice significantly reduced brain ASBT levels, BA levels, neuronal loss, and the extent of brain injury. Conversely, administering an ASBT activator (budesonide) elevated the average ASBT expressions, BA levels, and neuronal loss in the brain, significantly exacerbating brain injury. Therefore, this demonstrated through *in vivo* experiments that ASBT significantly regulates the effects of BA-induced neuronal loss and neurological impairment in mice.^61^ Our study identified the key role of hippocampal ASBT in mediating neuronal apoptosis and cognitive impairment in mice induced by DCA accumulation. Of note, dietary cholesterol upregulated hippocampal ASBT expression in obese mice, which may contribute to cognitive impairment. FXR, as a BA receptor, exerts a significant influence on cell apoptosis.^41,43^ Although we observed upregulation of hippocampal FXR of dietary cholesterol-fed mice, overexpression of FXR in the hippocampus had a limited impact on cognitive function, neuronal apoptosis, and caspase 3 cleavage in obese mice. The regulatory role of FXR in neuronal function has been reported. Blocking FXR signaling in the frontal cortex improves neurological decline in mice with acute liver injury.^25^ Neuron-specific knockout of FXR in the frontal cortex improves neurological decline in mice with chronic liver disease.^42^ This suggests that the regulatory role of FXR in neuronal function may vary across different diseases and brain regions. In our study, pharmacological inhibition of hippocampal caspase 3 cleavage ameliorated cognitive impairment and neuronal apoptosis induced by dietary cholesterol in obese mice.

In our study, dietary cholesterol increased DCA production by influencing gut microbiota, thereby promoting cognitive impairment in obese mice. The question remains whether cholesterol alone has a similar effect. In this study, dietary cholesterol alone did not significantly alter cognitive function in mice. We believe this may be due to two reasons: Firstly, dietary cholesterol alone had minor effects on hippocampal DCA levels, with only 1% significantly increasing DCA levels in the hippocampus. In comparison, DCA levels in the HFD + 0.2%, 0.5%, and 1% cholesterol groups were 2.0–2.6 times higher than in the HFD group and 4.3–5.5 times higher than in the Control group. Excessively high DCA levels may promote neuronal apoptosis and cognitive impairment in mice. Secondly, an HFD can induce neuronal apoptosis in the mouse brain.^62^ Therefore, under the influence of HFD, DCA is more likely to trigger neuronal apoptosis in the hippocampus. Importantly, dietary cholesterol and high-fat diet (HFD) may have a synergistic effect in causing cognitive impairment in mice, due to the following three reasons: First, under the combined effect of HFD and cholesterol, there is a greater increase in hippocampal DCA levels. Second, HFD alone can induce hippocampal cell apoptosis in mice, which may lead to increased neuronal apoptosis due to the DCA effects under HFD conditions. Third, while HFD did not significantly increase ASBT expression in the hippocampus, cholesterol elevated hippocampal ASBT expression in obese mice. Elevated ASBT expression could contribute to DCA accumulation in neuronal cells.

In summary, this study revealed a novel and convincing causal relationship between dietary cholesterol, gut microbiota, and its metabolites, and cognitive function in HFD-fed mice. Dietary cholesterol elevates the abundance of gut microbiota containing 7ɑ-dehydroxylase, including *Lachnospiraceae* bacterium, *Dorea* sp., and *Clostridium* sp., as well as the hippocampal expression of ASBT, leading to higher production of DCA and its accumulation in the hippocampus. This process induces neuronal apoptosis through caspase 3, ultimately contributing to cognitive impairment in HFD-fed mice. This study identifies DCA as a novel mediator of cognitive impairment and suggests that reducing bile acid levels may be an effective strategy for improving cognitive function. These mechanistic findings significantly advance our knowledge of the role of the diet-gut-brain axis in cognitive impairment, contributing to the development of dietary and microbial interventions to address this global health threat.

This study also has some limitations. Given that FMT provides a comprehensive approach to gut microbiota research, it does not definitively clarify the specific role of 7α-dehydroxylase-containing microbiota in cognitive impairment. Therefore, in our subsequent research, we plan to isolate and identify DCA-producing bacteria, such as *Clostridium* sp. and *Dorea* sp., and study their precise relationship with cognitive impairment in mice.

## Supplementary Material

Supplementary information 1.docx

Supplementary information 2.docx

Supplementary_information_3 clean.docx

## Data Availability

The metagenomic and transcriptomic data of this article are available in the NCBI repository (PRJNA1120581). The metabolomic data of this article are available in the National Genomics Data Center (PRJCA026860).

## Notes

All animal experiments were performed under all relevant ethical regulations for vertebrate animal research and were approved by the Experimental Animal Care and Use Committee of Zhejiang Chinese Medical University, number IACUC-20220328-24 and 20,210,927–16.
