# Supplementary material for: Dietary cholesterol impairs cognition via gut microbiota-derived deoxycholic acid in obese mice
Source: Gut Microbes. 2025 Jul 28;17(1):2537753. doi: 10.1080/19490976.2025.2537753 (PMC12309536; doi:10.1080/19490976.2025.2537753)
Supplement: Supplementary information 1.docx [file KGMI_A_2537753_SM9972.docx]

Supplementary information 1 Table S1. Ingredients of Control diet and HFD.

| Ingredients, g/kg | Control diet | HFD |
| --- | --- | --- |
| Protein (casein and L-cystine) | 141 | 177 |
| Carbohydrate (dextrin, sucrose, and corn starch) | 722 | 459 |
| Fat (soybean oil and lard) | 40 | 226 |
| Fiber (cellulose) | 50 | 62 |
| Mineral and vitamin mixture | 47 | 73 |
| Antioxidant (TBHQ) | 0.008 | 0.045 |
| Total, g | 1000 | 1000 |
| Energy, kcal/g | 3.6 | 4.5 |
| % kcal from fat | 10 | 45 |

Supplementary information 1 Table S2. Primer sequences utilized for real-time PCR.

| Gene name | Forward sequence (5’-3’) | Reverse sequence (5’-3’) |
| --- | --- | --- |
| Bax | AGACAGGGGCCTTTTTGCTAC | AATTCGCCGGAGACACTCG |
| Casp8 | AGATCCTGTGAATGGAACCTGGTAT | GTTCCTCCTGTCGTCTTTATTGCTC |
| Casp7 | AAGACGGAGTTGACGCCAAG | CCGCAGAGGCATTTCTCTTC |
| Casp3 | CTCGCTCTGGTACGGATGTG | TCCCATAAATGACCCCTTCATCA |
| Slc10a2 | TGGGTTTCTTCCTGGCTAGACT | TGTTCTGCATTCCAGTTTCCAA |
| Cyp46a1 | AGCCGCTATGAGCACATCC | CCATACTTCTTAGCCCAATCCAG |
| Cyp7a1 | CACCATTCCTGCAACCTTCTGG | ATGGCATTCCCTCCAGAGCTGA |
| Cyp8b1 | CATGAAGGCTGTGCGTGAGGAA | CATCACGCTGTCCAACACTGGA |
| Cyp27a1 | TCAGGAGACCATCGGCACCTTT | CCAGTCACTTCCTTGTGCAAGG |
| Cyp7b1 | CGGAAATCTTCGATGCTCCAAAG | GCTTGTTCCGAGTCCAAAAGGC |
| GAPDH | CATCACTGCCACCCAGAAGACTG | ATGCCAGTGAGCTTCCCGTTCAG |
