# Supplementary material for: Dietary cholesterol impairs cognition via gut microbiota-derived deoxycholic acid in obese mice
Source: Gut Microbes. 2025 Jul 28;17(1):2537753. doi: 10.1080/19490976.2025.2537753 (PMC12309536; doi:10.1080/19490976.2025.2537753)
Supplement: Supplementary information 2.docx [file KGMI_A_2537753_SM9971.docx]

Supplementary information 1

Materials and Methods

1. Transcriptome analysis

Total RNA was extracted from the hippocampal samples using Trizol reagent. The quantity and purity of the extracted RNA were assessed using a Bioanalyzer 2100 and RNA 6000 Nano LabChip Kit (Agilent, CA, USA, 5067-1511). High-quality RNA samples with an RNA integrity number > 7.0 were selected for library construction. mRNA was purified from the total RNA using Dynabeads Oligo (dT) (Thermo Fisher, CA, USA) with two rounds of purification. The purified mRNA was fragmented into short fragments using divalent cations under elevated temperature. The cleaved RNA fragments were reverse-transcribed to generate cDNA, which was then used to synthesize second-stranded DNAs. Adapters with custom Unique Molecular Identifiers were ligated to the fragmented DNA. Dual-index adapters were used for ligation, and size selection was performed using AMPureXP beads. After the heat-labile UDG enzyme (#m0280, NEB, USA) treatment of the U-labeled second-stranded DNAs, the ligated products were amplified by PCR. The final cDNA libraries, with an average insert size of 300±50 bp, were subjected to paired-end sequencing (PE150) on an Illumina Novaseq™ 6000 platform (LC-Bio Technology CO., Ltd., Hangzhou, China) according to the vendor's recommended protocol.

Reads were initially filtered using Cutadapt, followed by sequence quality verification using FastQC. Subsequently, reads from all samples were aligned to the mouse reference genome using the HISAT2 package. The mapped reads from each sample were then assembled using StringTie. Next, a comprehensive transcriptome was reconstructed by merging all transcriptomes using gffcompare software. Once the final transcriptome was generated, StringTie and ballgown were utilized to estimate expression levels for all transcripts and calculate the FPKM (fragments per kilobase of transcript per million mapped reads) value for mRNA expression abundance. Gene differential expression analysis was conducted using DESeq2 software between two distinct groups. Different expressed genes were then subjected to enrichment analysis of Gene Ontology (GO) functions and Kyoto Encyclopedia of Genes and Genomes (KEGG) pathways.

1. Metabolome analysis

The serum sample is added to a solution containing 400 μL of methanol/acetonitrile (1:1, v/v) with 0.02 mg/mL internal standard, and vortex mixed for 30 s. It is then sonicated at 4°C for 30 min. The sample is allowed to sit at -20 °C for 30 min to precipitate proteins. Subsequently, the sample is centrifuged at 13000 g for 15 min at 4 °C, and the supernatant is evaporated under nitrogen. The sample is then redissolved in 100 μL of acetonitrile/water (1:1, v/v), sonicated at 4 °C for 5 min, and centrifuged at 13000 g for 10 min at 4 °C. The supernatant obtained was utilized for analysis.

The LC-MS/MS analysis of the sample was performed using an UHPLC-Q Exactive HF-X system (Thermo Fisher Scientific, Waltham, MA, USA), featuring an ACQUITY HSS T3 column (100 mm × 2.1 mm i.d., 1.8 μm; Waters, USA), at Majorbio Bio-Pharm Technology Co. Ltd. (Shanghai, China). The mobile phases comprised 0.1% formic acid in water:acetonitrile (95:5, v/v) (solvent A) and 0.1% formic acid in acetonitrile:isopropanol:water (47.5:47.5, v/v) (solvent B). In positive ion mode separation gradient: From 0 to 3 min, mobile phase B increased from 0% to 20%; from 3 to 4.5 min, mobile phase B increased from 20% to 35%; from 4.5 to 5 min, mobile phase B increased from 35% to 100%; from 5 to 6.3 min, mobile phase B was held at 100%; from 6.3 to 6.4 min, mobile phase B decreased from 100% to 0%; from 6.4 to 8 min, mobile phase B was held at 0%. In negative ion mode separation gradient: From 0 to 1.5 min, mobile phase B increased from 0 to 5%; from 1.5 to 2 min, mobile phase B increased from 5% to 10%; from 2 to 4.5 min, mobile phase B increased from 10% to 30%; from 4.5 to 5 min, mobile phase B increased from 30% to 100%; from 5 to 6.3 min, mobile phase B was maintained at 100%; from 6.3 to 6.4 min, mobile phase B decreased from 100% to 0%; from 6.4 to 8 min, mobile phase B was maintained at 0%. The flow rate was set at 0.40 mL/min, and the column temperature was maintained at 40°C. MS conditions: The mass spectrometric data were acquired using a UHPLC-Q Exactive HF-X Mass Spectrometer equipped with an electrospray ionization (ESI) source operating in both positive and negative modes. The optimized settings were as follows: source temperature set at 425 °C; sheath gas flow rate at 50 arb; Aux gas flow rate at 13 arb; ion-spray voltage floating (ISVF) set at -3500 V in negative mode and 3500V in positive mode, respectively; Normalized collision energy, with a rolling range of 20-40-60V for MS/MS. Full MS resolution was set at 60000, and MS/MS resolution at 7500. Data acquisition was conducted using the Data Dependent Acquisition mode, covering a mass range of 70-1050 m/z.

The pretreatment of LC/MS raw data was performed by Progenesis QI (Waters Corporation，Milford, USA) software. The metabolites were identified by searching database, and the main databases were the HMDB (http://www.hmdb.ca/), Metlin (https://metlin.scripps.edu/) and Majorbio Database. The data matrix was pre-processed, as follows: At least 80% of the metabolic features detected in any set of samples were retained. After filtering, for specific samples with metabolite levels below the lower limit of quantification, the minimum metabolite value was estimated and each metabolic signature was normalized to the sum. To reduce the errors caused by sample preparation and instrument instability, the response intensities of the sample mass spectrometry peaks were normalized using the sum normalization method, to obtain the normalized data matrix. Meanwhile, the variables of QC samples with relative standard deviation (RSD) > 30% were excluded and log10 logarithmicized, to obtain the final data matrix for subsequent analysis.

Then, the R package “ropls” (Version 1.6.2) was used to perform least partial squares discriminant analysis (PLS-DA). The metabolites with VIP>1, p<0.05 were determined as significantly different metabolites based on the Variable importance in the projeciton (VIP) obtained by the PLS-DA model and the p-value generated by student’s t test.

1. Metagenome analysis

Total genomic DNA was extracted from fecal samples using the E.Z.N.A. Stool DNA Kit (Omega Bio-tek, Norcross, GA, U.S.) according to manufacturer’s instructions. Concentration and purity of extracted DNA was determined with NanoDrop2000. DNA extract quality was checked on 1% agarose gel.

DNA extract was fragmented to an average size of about 400 bp using Covaris M220 (Gene Company Limited, China) for paired-end library construction. Paired-end library was constructed using NEXTFLEX Rapid DNA-Seq (Bioo Scientific, Austin, TX, USA). Adapters containing the full complement of sequencing primer hybridization sites were ligated to the blunt-end of fragments. Paired-end sequencing was performed on Illumina Novaseq 6000 (Illumina Inc., San Diego, CA, USA) at Majorbio Bio-Pharm Technology Co., Ltd. (Shanghai, China).

The data were analyzed on the free online platform of Majorbio Cloud Platform (www.majorbio.com). Briefly, the paired-end Illumina reads were trimmed of adaptors, and low-quality reads (length<50 bp or with a quality value <20 or having N bases) were removed by fastp (https://github.com/OpenGene/fastp, version 0.20.0). Reads were aligned to the mouse genome by BWA (http://bio-bwa.sourceforge.net, version 0.7.9a) and any hit associated with the reads and their mated reads were removed. Metagenomics data were assembled using MEGAHIT (<https://github.com/voutcn/megahit>, version 1.1.2), which makes use of succinct de Bruijn graphs . Contigs with with a length ≥ 300 bp were selected as the final assembling result, and then the contigs were used for further gene prediction and annotation.

Open reading frames (ORFs) from each assembled contig were predicted using Prodigal/MetaGene (http://metagene.cb.k.u-tokyo.ac.jp/). The predicted ORFs with a length ≥ 100 bp were retrieved and translated into amino acid sequences using the NCBI translation table (http://www.ncbi.nlm.nih.gov/Taxonomy/taxonomyhome.html/index.cgi?chapter=tgencodes#SG1.

A non-redundant gene catalog was constructed using CD-HIT (http://www.bioinformatics.org/cd-hit/, version 4.6.1) with 90% sequence identity and 90% coverage. High-quality reads were aligned to the non-redundant gene catalogs to calculate gene abundance with 95% identity using SOAPaligner (http://soap.genomics.org.cn/, version 2.21).

[Representative sequences](http://en.wikipedia.org/wiki/Representative_sequences) of non-redundant gene catalog were aligned to NR database with an e-value cutoff of 1e^-5^ using Diamond (http://www.diamondsearch.org/index.php, version 0.8.35) for taxonomic annotations. The statistical analysis of the data was conducted using the EasyAmplicon.
