# Supplementary material for: Dietary cholesterol impairs cognition via gut microbiota-derived deoxycholic acid in obese mice
Source: Gut Microbes. 2025 Jul 28;17(1):2537753. doi: 10.1080/19490976.2025.2537753 (PMC12309536; doi:10.1080/19490976.2025.2537753)
Supplement: Supplementary_information_3 clean.docx [file KGMI_A_2537753_SM9970.docx]

**Supplementary Figures**

**Fig. S1.** The intake of cholesterol is significantly negatively correlated with cognitive levels. (A) Effects of dietary cholesterol on the food intake of mice. Ten mice were kept in three cages, and the feed consumption of the mice in each cage was recorded. (B) Linear regression analysis of dietary cholesterol intake and cognitive function. (C) Effects of dietary cholesterol alone on the Body weight change and food intake of mice fed a control diet (n=10). (D) Percentage of alteration and total arm entries in the Y-maze test (n=10). (E) Discrimination rate and total exploration time in the novel object recognition test (n=10). Statistical analysis was performed using a one-way ANOVA followed by Dunnett post hoc test. * *p* <0.05, ** *p* <0.01, *** *p* <0.001.


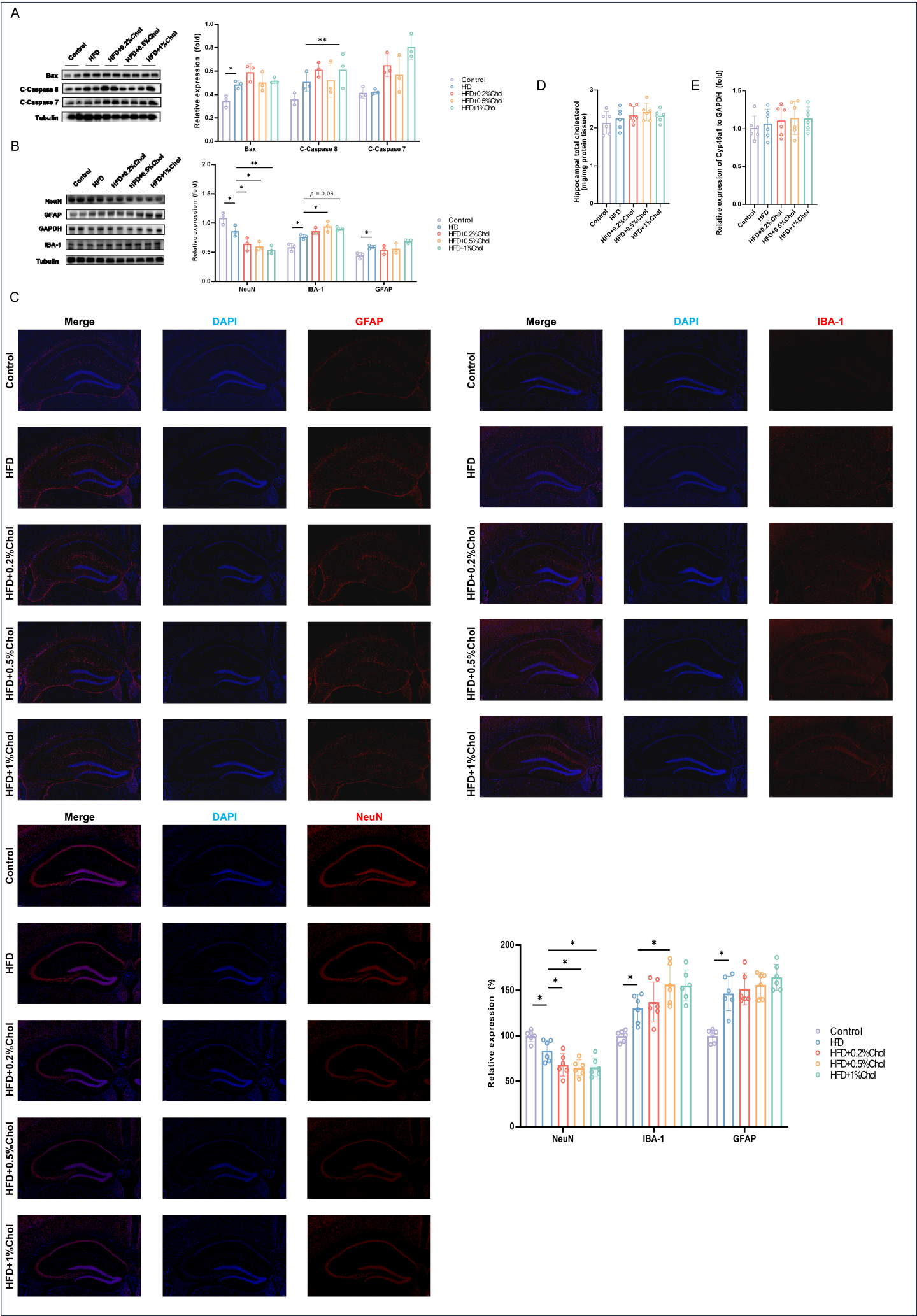


**Fig. S2.** Effects of dietary cholesterol on apoptosis-related proteins and cholesterol level in hippocampus of HFD-fed mice. (A) Effects of dietary cholesterol on hippocampal Bax, C-Caspase 7, and C-Caspase 8 in HFD-fed mice (n=3). (B) Effects of dietary cholesterol on hippocampal NeuN, IBA-1, and GFAP in HFD-fed mice (n=3). (C) Immunofluorescence analysis of hippocampal NeuN, IBA-1, and GFAP (n=6). (D) Hippocampal levels of total cholesterol (n=6). (E) Relative expression of Cyp46a1(n=6). Statistical analysis was performed using a one-way ANOVA followed by Dunnett post hoc test. * p <0.05, ** p <0.01, *** p <0.001.


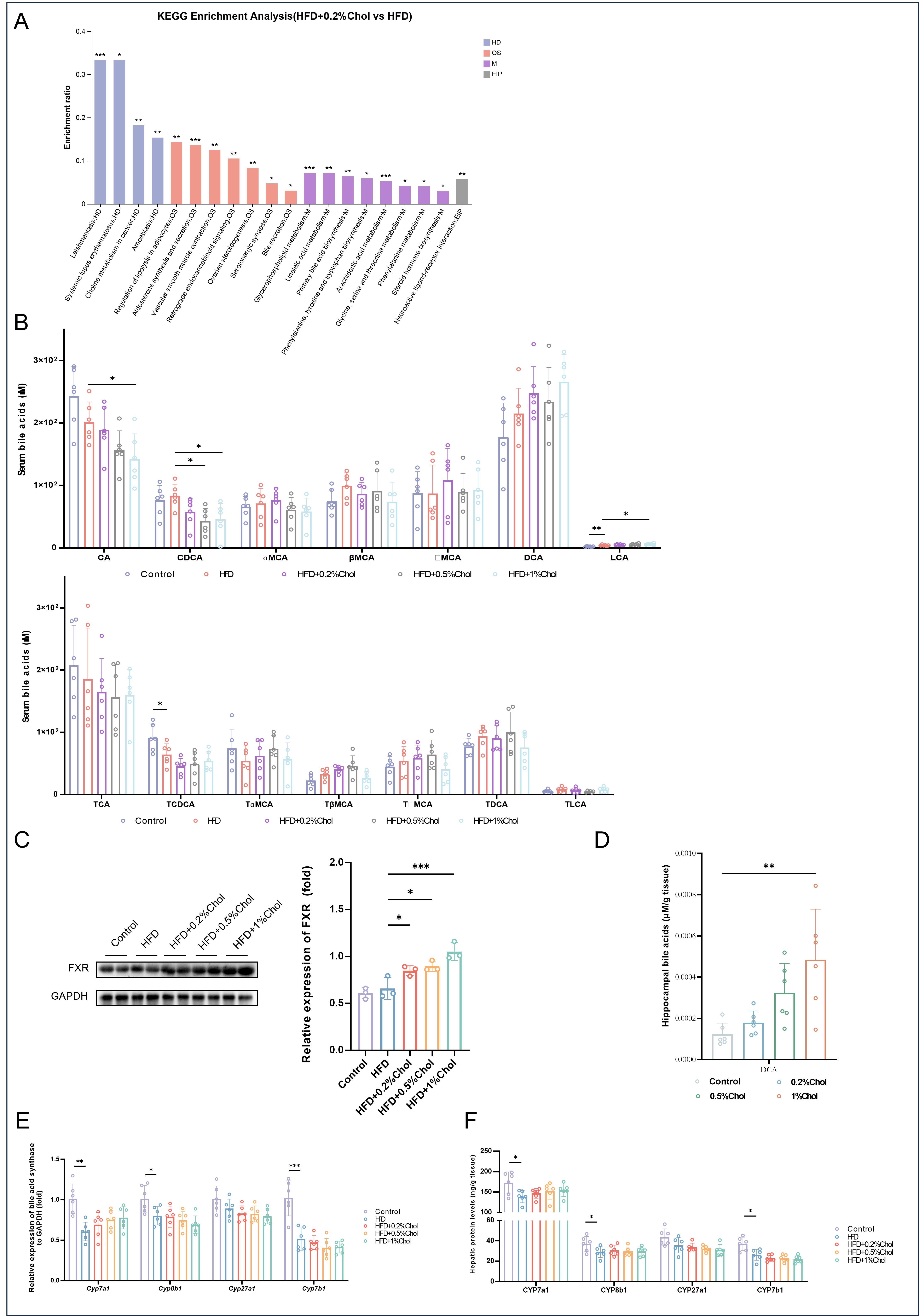


**Fig. S3**. Dietary cholesterol affected the serum metabolome and hippocampal FXR expression but did not impact bile acid synthesis enzymes. (A) KEGG enrichment analysis. (B) Serum levels of major unconjugated and taurine-conjugated bile acids in mice (n=6). (C) Protein expression of FXR (n=6). (D) Effects of dietary cholesterol alone on hippocampal DCA levels in mice fed a control diet.(n=6). (E) mRNA expression of bile acid synthase (n=6). (F) Hepatic protein levels of bile acid synthase (n=6). Statistical analysis was performed using a one-way ANOVA followed by Dunnett post hoc test. * *p* <0.05, ** *p* <0.01, *** *p* <0.001.

**Fig. S4**. Dietary cholesterol altered the composition of the gut microbiota in HFD-fed mice. (A) PCoA analysis of fecal microbiota profile at the genus level in the HFD and HFD+0.2%Chol-fed mice (n=6). (B) ɑ-diversity of fecal microbiota at the species level (n=6). (C) Effect of 0.2% cholesterol on the relative abundance of microbiota at the phylum level in HFD-fed mice. (D) The significantly different microbiota at the phylum level. For the differences of microbiota, Wilcoxon rank-sum test (two-tailed test) was employed. For the other data, an unpaired t-test was used.

**Fig. S5**. Dietary cholesterol altered the composition of the gut microbiota in HFD-fed mice. (A) Effect of 0.2% cholesterol on the relative abundance of microbiota at the genus level in HFD-fed mice. (B) The significantly different microbiota at the genus level. (C) Effect of 0.2% cholesterol on the relative abundance of microbiota at the species level in HFD-fed mice. (D) The significantly different microbiota at the species level. For the differences of microbiota, Wilcoxon rank-sum test (two-tailed test) was employed.

**Fig. S6** Under the influence of dietary cholesterol, the gut microbiota of HFD-fed mice tends to shift towards a competitive relationship. (A) Topological properties of murine gut microbial networks. (B) Network of the HFD group and the HFD+0.2%Chol group based on Spearman’s correlation analysis. (C) Weighted degree of co-occurrence network nodes.

**Fig. S7**. Antibiotics reduced the abundance of gut microbiota in mice, while FMT restored it. (A) Fecal microbiota level after the Abx (B) Fecal microbiota level after the FMT. Statistical analysis was performed using two-way ANOVA followed by Tukey post hoc test. In (A), the 16s rDNA copies of each group on day 0 were compared with those on days 2, 4, 6, 8, and 10. In (B), the 16s rDNA copies of each group on day 0 were compared with those on days 3, 5, and 7. * *p* <0.05, ** *p* <0.01, *** *p* <0.001.


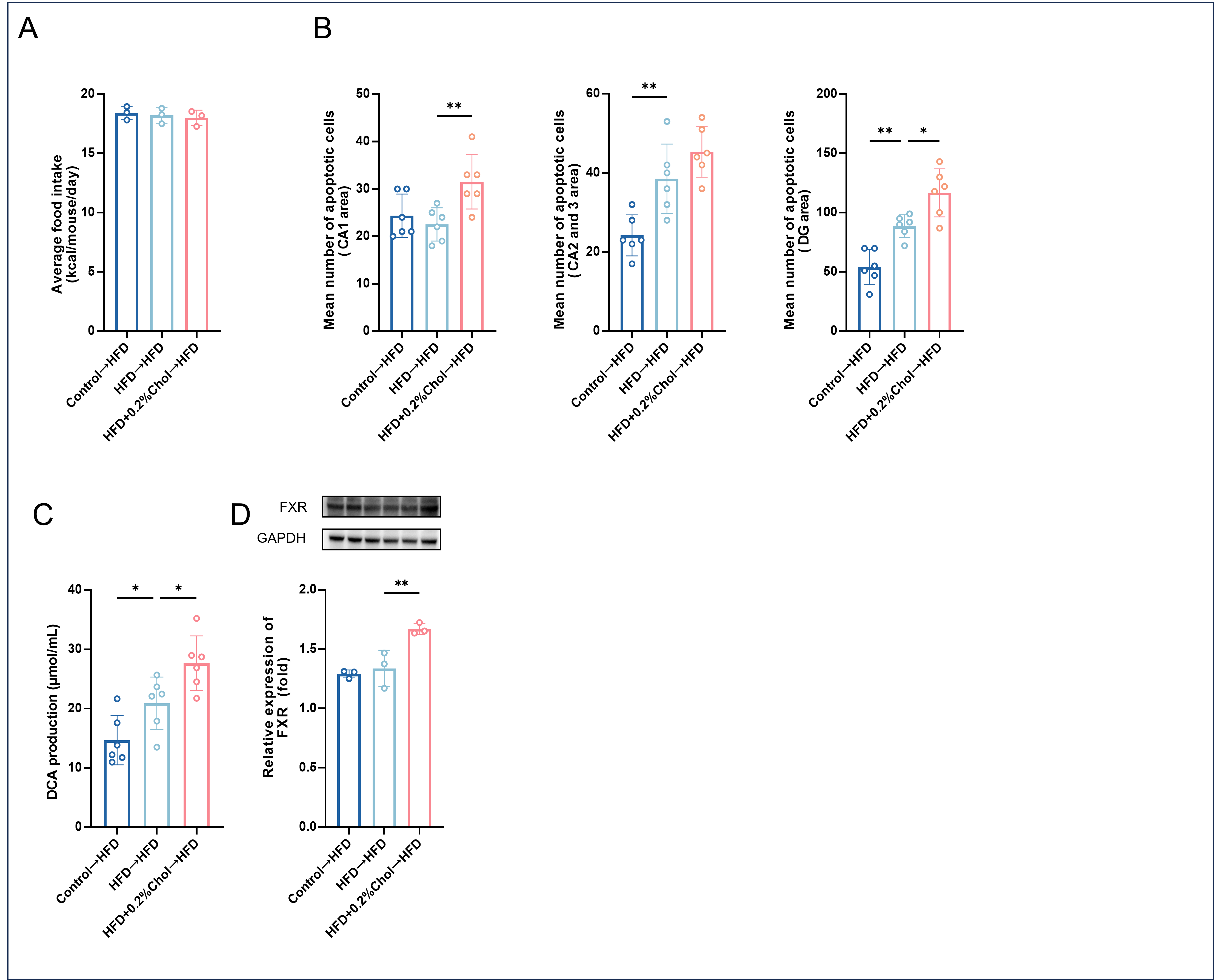


**Fig. S8.** FMT increased the ability of fecal bacteria to produce DCA in mice and enhanced FXR expression in the hippocampus. (A) Average food intake (n=3). (B) The quantification of apoptotic cells in different hippocampal area (n=6). (C) *In vitro* assessment of DCA production by fecal bacteria (n=6). (D) Protein expression of FXR (n=3). Statistical analysis was performed using one-way ANOVA followed by Dunnett post hoc test. * *p* <0.05, ** *p* <0.01, *** *p* <0.001.


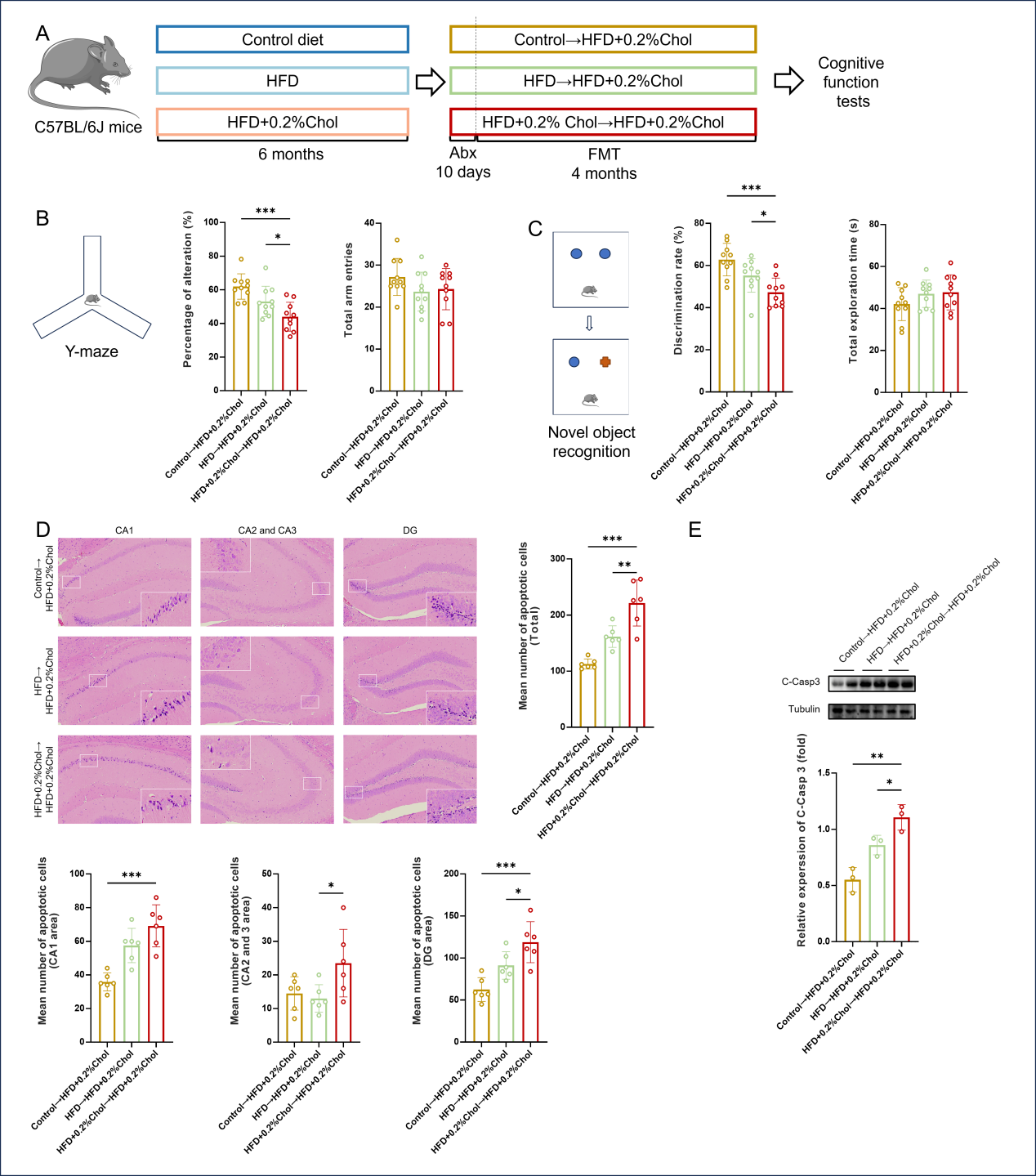


Fig. S9. Transplanting fecal microbiota from the HFD group mice to the HFD+0.2%Chol group mice improve cognitive function. (A) Experimental design. (B) Percentage of alteration and total arm entries in the Y-maze test (n=10). (C) Discrimination rate and total exploration time in the novel object recognition test (n=10). (D) Representative images of hippocampus H&E staining (magnification 100×) and the quantification of apoptotic cells in the hippocampus (n=6). (E) Protein expression of cleaved-Caspase-3 (n=3). Statistical analysis was performed using one-way ANOVA followed by Dunnett post hoc test. * *p* <0.05, ** *p* <0.01, *** *p* <0.001.

**Fig. S10.** Effects of CHY and DCA on hippocampal neuronal apoptosis in mice. Effects of CHY on the (A) body weight change, (B) average food intake, and (C) total bile acids in feces, serum, and brain of HFD+0.2% cholesterol-fed mice (n=10). (D) Effects of CHY on the number of apoptotic cells in different regions of the mouse hippocampus (n=6). (E) Effects of CHY on the C-Caspase 3 in hippocampus (n=3). Effects of DCA on the (F) body weight change and (G) average food intake in HFD-fed mice (n=10). (H) Effects of DCA on the number of apoptotic cells in different regions of the mouse hippocampus (n=6). (I) Effects of DCA on the C-Caspase 3 in hippocampus (n=3). Statistical analysis was performed using an unpaired t-test. * *p* <0.05, ** *p* <0.01, *** *p* <0.001.


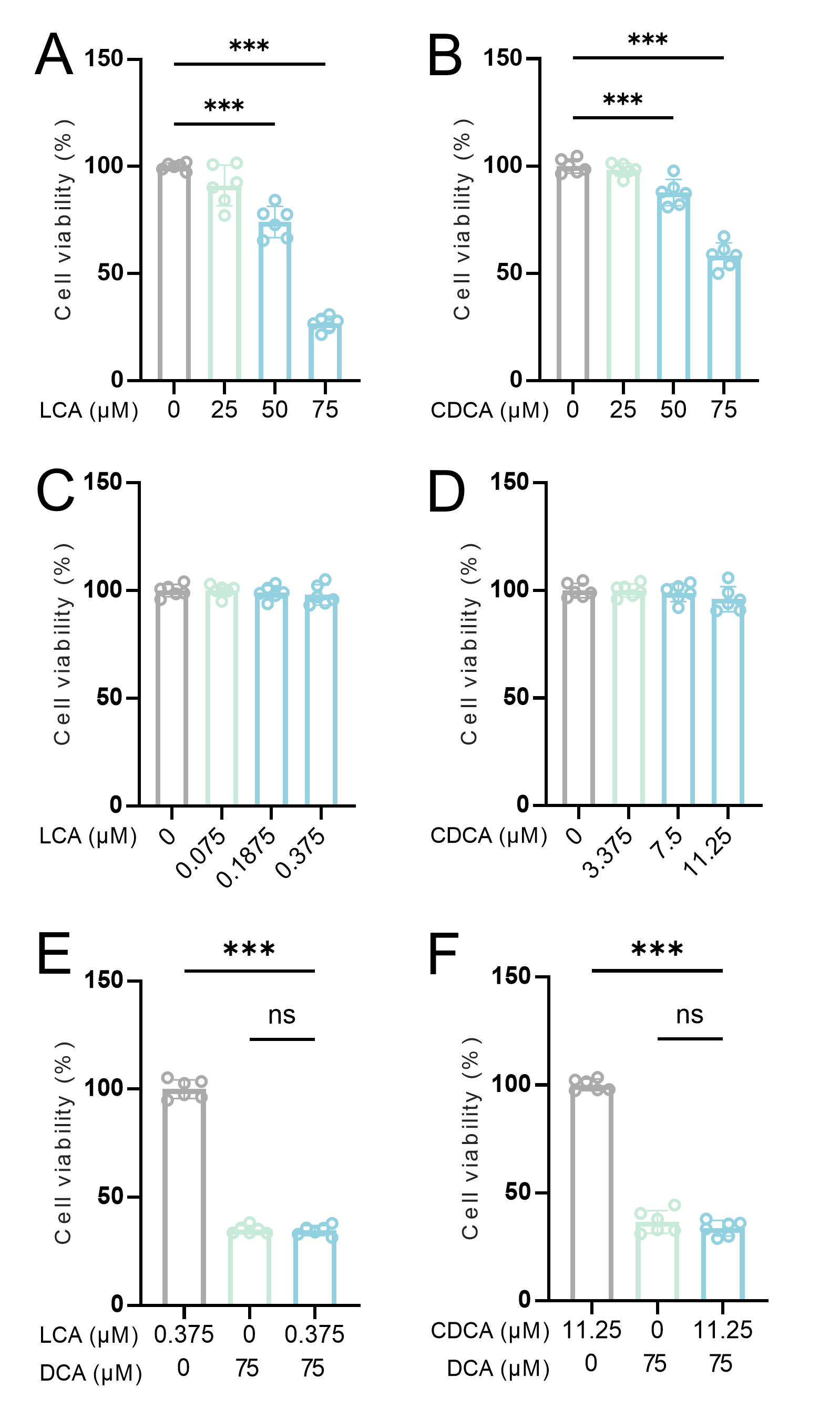


**Fig. S11.** The cytotoxicity of LCA and CDCA on SH-SY5Y cell. (A) Cytotoxicity of LCA at 0-75 μM. (B) Cytotoxicity of CDCA at 0-75 μM. (C) Cytotoxicity of LCA at 0-0.375 μM. (D) Cytotoxicity of CDCA at 0-11.25 μM. (E) Cytotoxicity of the combination of LCA and DCA. (F) Cytotoxicity of the combination of CDCA and DCA. Statistical analysis was performed using one-way ANOVA followed by Dunnett post hoc test. * *p* <0.05, ** *p* <0.01, *** *p* <0.001.

**Fig. S12.** Guggulsterone and Z-DEVD-FMK improved cell apoptosis induced by DCA. (A) Cell viability after 0.5-25 μM DEX treatments in SH-SY5Y for 96 h (n=6). (B) Cell viability after 1-50 μM GSK treatments in SH-SY5Y for 96 h (n=6). (C) TUNEL density after Gugg (1-15μM) and DCA (75 μM) co-treatments in SH-SY5Y for 24 h (n=6). (D) Caspase 3 activity after Gugg (1-15μM) and DCA (75 μM) co-treatments in SH-SY5Y for 24h (n=6). (E) TUNEL density after ZDF (25-100μM) and DCA (75 μM) co-treatments in SH-SY5Y for 24 h (n=6). Statistical analysis was performed using one-way ANOVA followed by Dunnett post hoc test. * *p* <0.05, ** *p* <0.01, *** *p* <0.001. DEX, dexamethasone; GSK, GSK2330672; Gugg, guggulsterone; ZDF, Z-DEVD-FMK.

**Fig. S13**. Overexpression of FXR has a limited impact on cognitive function in HFD-fed mice. (A) Experimental design. (B) Protein expression of FXR (n=3). (C) Body weight change and average food intake (n=10). (D) Percentage of alteration and total arm entries in the Y-maze test (n=10). (E) Discrimination rate and total exploration time in the novel object recognition test (n=10). (F) Representative images of hippocampus H&E staining (magnification 100×). (G) The quantification of apoptotic cells in the hippocampus (n=6). (H) Protein expression of cleaved Caspase 3 (n=3). Statistical analysis was performed using an unpaired t-test between the AAV-Control and AAV-*Nr1h4*-treated groups. * *p* <0.05, ** *p* <0.01, *** *p* <0.001.

**Fig. S14**. Inhibition of caspase 3 improves the pro-cognitive impairment effects of dietary cholesterol in HFD-fed mice. (A) Experimental design. (B) Body weight change and average food intake (n=10). (C) Percentage of alteration and total arm entries in the Y-maze test (n=10). (D) Discrimination rate and total exploration time in the novel object recognition test (n=10). (E) Representative images of hippocampus H&E staining (magnification 100×). (F) The quantification of apoptotic cells in the hippocampus (n=6). (G) Protein expression of cleaved-Caspase-3 (n=3). Statistical analysis was performed using two-way ANOVA followed by Tukey post hoc test. * *p* <0.05, ** *p* <0.01, *** *p* <0.001.
